# Rec8 Phosphorylation by Casein Kinase 1 and Cdc7-Dbf4 Kinase Regulates Cohesin Cleavage by Separase during Meiosis

**DOI:** 10.1016/j.devcel.2010.01.014

**Published:** 2010-03-16

**Authors:** Vittorio L. Katis, Jesse J. Lipp, Richard Imre, Aliona Bogdanova, Elwy Okaz, Bianca Habermann, Karl Mechtler, Kim Nasmyth, Wolfgang Zachariae

**Affiliations:** 1 Department of Biochemistry, University of Oxford, South Parks Road, Oxford, OX1 3QU, UK; 2 Max Planck Institute of Molecular Cell Biology and Genetics, Pfotenhauerstrasse 108, 01307 Dresden, Germany; 3 Research Institute of Molecular Pathology (IMP), Dr. Bohr-Gasse 7, A-1030 Vienna, Austria

**Keywords:** CELLBIO

## Abstract

During meiosis, two rounds of chromosome segregation after a single round of DNA replication produce haploid gametes from diploid precursors. At meiosis I, maternal and paternal kinetochores are pulled toward opposite poles, and chiasmata holding bivalent chromosomes together are resolved by cleavage of cohesin's α-kleisin subunit (Rec8) along chromosome arms. This creates dyad chromosomes containing a pair of chromatids joined solely by cohesin at centromeres that had resisted cleavage. The discovery that centromeric Rec8 is protected from separase during meiosis I by shugoshin/MEI-S332 proteins that bind PP2A phosphatase suggests that phosphorylation either of separase or cohesin may be necessary for Rec8 cleavage. We show here that multiple phosphorylation sites within Rec8 as well as two different kinases, casein kinase 1δ/ɛ (CK1δ/ɛ) and Dbf4-dependent Cdc7 kinase (DDK), are required for Rec8 cleavage and meiosis I nuclear division. Rec8 with phosphomimetic mutations is no longer protected from separase at centromeres and is cleaved even when the two kinases are inhibited. Our data suggest that PP2A protects centromeric cohesion by opposing CK1δ/ɛ- and DDK-dependent phosphorylation of Rec8.

## Graphical abstract

**Graphical Abstract:**

**Figure:**
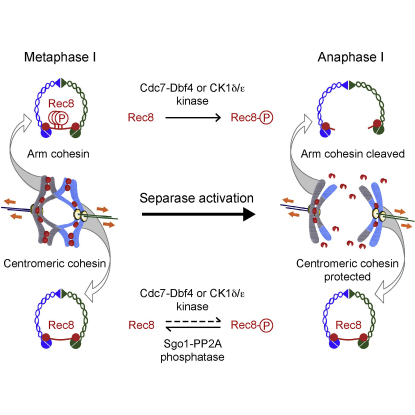

**Highlights:**

► Cleavage of cohesin at meiosis I requires phosphorylation of its Rec8 subunit ► Nonphosphorylatable Rec8 blocks the resolution of chiasmata in a dominant manner ► Separase preferentially cleaves Rec8 phosphorylated by the kinases DDK and CK1 ► PP2A protects centromeric cohesin at meiosis I by local dephosphorylation of Rec8

## Introduction

During mitosis, a multisubunit complex called cohesin entraps sister chromatids within a large proteinaceous ring and thereby holds them together from their creation during S phase until their disjunction to opposite halves of the cell at anaphase. By resisting the tendency of microtubules to pull sister chromatids apart during metaphase, cohesin creates the tension thought to be necessary to stabilize selectively amphitelic microtubule-kinetochore attachments, which connect sister chromatids to opposite spindle poles. Sister chromatids are eventually disjoined by separase, a thiol protease that cleaves cohesin's α-kleisin subunit Scc1/Rad21, opens cohesin's tripartite ring, and releases sister chromatids from their embrace. Separase is kept inactive for most of the cell cycle by the binding of an inhibitory chaperone known as securin (Pds1 in yeast), whose destruction at the hands of a ubiquitin ligase called the anaphase-promoting complex (APC/C) only takes place after all chromosomes have bioriented, i.e., attached to microtubules in an amphitelic manner.

During meiosis, two rounds of chromosome segregation (meiosis I and II) without an intervening round of DNA replication produce haploid gametes from diploid progenitors. This is made possible by the lack of DNA replication between the meiotic divisions and by three key features of meiosis I (Petronczki et al., 2003). First, reciprocal recombination between homologous nonsister chromatids produces chiasmata that hold all four homologous chromatids together, thereby forming bivalent chromosomes. Second, monopolin proteins prevent the traction of sister kinetochores toward opposite spindle poles. As a consequence, the tension necessary to stabilize microtubule-kinetochore interactions is generated by pulling maternal and paternal sister kinetochore pairs (and not sisters) in opposite directions. Third, separase cleaves cohesin along chromosome arms, but not at centromeres. This resolves chiasmata and triggers the disjunction to opposite poles of dyad chromosomes containing a pair of chromatids joined solely at their centromeres by cohesin that had resisted cleavage. The latter is essential for the subsequent amphitelic attachment of sister kinetochores during meiosis II and is eventually destroyed by a second round of separase activity. The trigger for both meiotic divisions is thought to be identical to that during mitosis, namely, the ubiquitinylation of securin (and cyclin B) by the APC/C, producing a burst of separase activity that cleaves cohesin's meiosis-specific α-kleisin subunit Rec8 (Buonomo et al., 2000; Kitajima et al., 2003; Kudo et al., 2006).

The persistence of cohesin at centromeres after the first meiotic division explains the unusual ability of meiotic cells to undergo a second round of chromosome segregation (meiosis II) without a preceding round of DNA replication during which cohesion is normally established (Nasmyth and Haering, 2005). What determines the different fates of cohesin at centromeres and on chromosome arms after the first wave of separase activity? Recent work has established that orthologs of the *Drosophila* MEI-S332 protein, called shugoshins, are required (Katis et al., 2004a; Kerrebrock et al., 1995; Kitajima et al., 2004; Marston et al., 2004; Rabitsch et al., 2004). Shugoshins are thought to control Rec8 cleavage by recruiting to kinetochores a PP2A phosphatase complex with a regulatory subunit of the B′ type (Rts1 in yeast) (Kitajima et al., 2006; Riedel et al., 2006). Crucially, a budding yeast shugoshin mutant defective solely in the binding to PP2A fails to protect centromeric Rec8 in meiosis I (Xu et al., 2009).

The finding that shugoshins protect centromeric cohesin by recruiting PP2A implies that the phosphorylation of some protein is necessary for Rec8 cleavage. Candidates include Rec8 itself and separase. A clue that Rec8 might be PP2A's target is the finding that yeast cells expressing Scc1 instead of Rec8 during meiosis fail to protect centromeric cohesin, at least when recombination has been eliminated (Tóth et al., 2000). If so, which kinase phosphorylates Rec8? In mitotic yeast cells, cohesin cleavage is promoted through the phosphorylation of Scc1 by polo-like kinase (PLK, Cdc5 in yeast) (Alexandru et al., 2001; Hornig and Uhlmann, 2004), which also participates in the phosphorylation of Rec8 (Clyne et al., 2003; Lee and Amon, 2003). Surprisingly, replacement by alanine of Rec8 residues thought to be phophorylated by Cdc5 has little or no effect on the kinetics of cohesin cleavage at meiosis I (Brar et al., 2006). Either Cdc5 is not the kinase responsible for promoting Rec8 cleavage or separase might after all be PP2A's real target. To address these key issues, which are fundamental to our understanding of meiosis, we have analyzed Rec8 phosphorylation without making any assumption about the kinase responsible. We show that substitution of 24 phosphorylated residues by alanine greatly hinders cleavage, whereas substitution of a subset of these with aspartate, mimicking the effects of phosphorylation, causes precocious loss of sister centromere cohesion. In addition, we show that casein kinase 1δ/ɛ (CK1δ/ɛ, Hrr25 in yeast) and Dbf4-dependent Cdc7 kinase (DDK), and not Cdc5, are essential for Rec8 cleavage. Our data suggest that shugoshins protect centromeric cohesin by opposing Rec8's phosphorylation by CK1δ/ɛ and DDK.

## Results

### Identification of Rec8 Phosphorylation Sites

A tandem affinity purification (TAP) tag was used to isolate Rec8 from extracts of diploid yeast cells arrested in metaphase of meiosis I. Purified proteins were analyzed by gel electrophoresis (see Figure S1A available online) or digested in solution with different proteases for mass spectrometric peptide identification. In addition to Rec8, we detected the cohesin subunits Smc1, Smc3, Scc3, and Pds5 (Table S1). Consistent with previous work (Matos et al., 2008; Petronczki et al., 2006), Rec8 was associated with the protein kinases Cdc5/PLK and Hrr25/CK1δ/ɛ. Interestingly, Rec8 also copurified with the meiosis-specific recombination proteins Dmc1 and Hop1.

**Figure 1:**
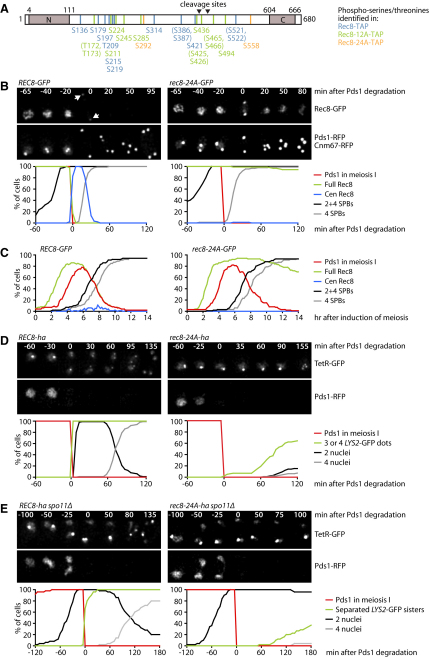
Rec8 Phosphorylation Is Required for Chiasmata Resolution upon APC/C Activation (A) Phosphorylated serines and threonines identified in purified Rec8-TAP (blue), Rec8-12A-TAP (green), and Rec8-24A-TAP (orange). Regions conserved between α-kleisins are gray. See also Figure S1 and Table S1. (B and C) Live imaging of meiotic *REC8-GFP* (Z12781) and *rec8-24A-GFP* (Z12782) cells containing Cnm67-RFP at SPBs and Pds1-RFP (fluorescence detectable only in meiosis I). (B) Top: time-lapse series. Arrows mark centromeric Rec8. Bottom: the presence of Pds1 (meiosis I, red), Rec8 (entire chromatin, green; centromeric, blue), two or four SPBs (black), and four SPBs (gray) was quantified every 5 min in 100 cells, in which Pds1 degradation was set to t = 0. (C) Parameters listed in (B) were quantified every 10 min after the induction of meiosis (t = 0) in 100 cells. See also Figures S2A and S2B. (D) Live imaging of meiotic *REC8-ha* (Z15617) and *rec8-24A-ha* (Z13861) cells containing Pds1-RFP, TetR-GFP, and homozygous *LYS2-tetO*. TetR-GFP labels the nucleus (diffuse signal) and all *LYS2* loci (dots). Top: time-lapse series. Bottom: the presence of Pds1 (meiosis I, red), three or four *LYS2*-GFP dots (loss of arm cohesion, green), two nuclei (black), and four nuclei (gray) was quantified every 5 min in 100 cells, in which Pds1 degradation was set to t = 0. (E) Live imaging of meiotic *REC8-ha spo11Δ* (Z15789) and *rec8-24A-ha spo11Δ* (Z15790) cells containing Pds1-RFP, TetR-GFP, and heterozygous *LYS2-tetO*. Meiosis was analyzed as in (D), except loss of arm cohesion is indicated by two *LYS2*-GFP dots (green).

Analysis of Rec8 peptides covering 95% of the sequence revealed phosphorylation of eight serine or threonine residues. Two Ser-Ser sequences carried a phosphate group on either one of the two residues (Figure 1A, blue residues; Figure S1A). If Rec8 phosphorylation were important for its cleavage by separase, mutation of these to alanine, which cannot be phosphorylated, should block the meiosis I division. However, substitution of all 12 residues has little effect on the meiotic progression of homozygous *rec8-12A* cells (data not shown). There are two possible explanations for this finding: either the phosphorylation of Rec8 is unimportant, or additional residues are phosphorylated when primary sites are mutated. To investigate the latter explanation, we mapped phosphorylation sites within Rec8-12A purified from meiotic cells. This revealed nine phosphorylated residues and one phosphate group in each of three Ser-Ser or Thr-Thr sequences (Figure 1A, green residues; Figure S1B). These additional residues were also mutated to alanine, and the resulting Rec8-24A protein was subjected to a third round of phosphosite mapping, which uncovered two more phosphorylated residues (Figure 1A, orange residues; Figure S1C). Interestingly, all 26 phosphorylation sites are located within the central region of Rec8, which is poorly, if at all, conserved among kleisins. Of these phosphorylation sites, nine were not identified in a previous analysis of Rec8 (Brar et al., 2006).

### Nonphosphorylatable Rec8 Persists on Chromatin and Blocks Chiasmata Resolution

To analyze the consequences of preventing Rec8's phosphorylation, we used live imaging to observe GFP-tagged versions of Rec8 or Rec8-24A together with Pds1-RFP and the spindle pole body (SPB) component Cnm67-RFP (Figure 1B). In addition, we measured protein levels by immunoblot analysis of protein extracts (Figure S2A). Most of the Rec8-GFP disappears from chromatin at the same time as Pds1-RFP destruction and SPB segregation, after which Rec8-GFP persists exclusively as faint “centromeric” clusters associated with each spindle pole until disappearing from view as centromeres disperse around the time spindle poles separate during meiosis II. Strikingly, Rec8-24A-GFP persists throughout the nucleus long after Pds1 destruction and remains at high levels even after SPB reduplication and separation in meiosis II (Figure 1B). Homozygous *rec8-24A-GFP* cells separate SPBs, express proteins required to enter metaphase I, and degrade Pds1-RFP with kinetics comparable to that of *REC8-GFP* cells (Figure 1C; Figure S2A). The Rec8-24A protein does not, therefore, cause a significant delay in entry into and progression through meiosis I. Rec8-24A remains at high levels beyond meiosis I also in cells lacking Sgo1, an inhibitor of cohesin removal from chromatin (Figure S2B). We conclude that the nonphosphorylatable Rec8-24A protein resists removal from chromatin and degradation at the metaphase I-to-anaphase I transition.

To investigate the role of Rec8 phosphorylation in meiotic chromosome segregation, we imaged homozygous *REC8-ha* and *rec8-24A-ha* strains containing Pds1-RFP and a *tet* repressor-GFP fusion (TetR-GFP), which binds to *tet* operators integrated at *LYS2* on the arms of both chromosome II homologs (Figure 1D). After S phase, TetR-GFP bound to *tetO* marks all four *LYS2* sister sequences, and the free fraction labels the nucleus. Recombination causes the marked *LYS2* loci to coalesce into a single GFP dot during prophase I. In wild-type cells, the degradation of Pds1-RFP triggers loss of sister chromatid cohesion on chromosome arms: the *LYS2*-GFP dot splits into two pairs of GFP foci, which segregate into the two daughter nuclei resulting from the meiosis I division. Neither the splitting of the *LYS2*-GFP dot nor nuclear division occurs upon Pds1-RFP destruction in *rec8-24A* cells. To address whether *rec8-24A* hinders the resolution of chiasmata, we eliminated Spo11, the endonuclease that initiates recombination. Crucially, deletion of *SPO11* restores the meiosis I, but not the meiosis II, division in *rec8-24A* cells (Figure 1E). This also implies that sister centromeres are properly mono-orientated at meiosis I in these cells. We conclude that separase activation fails to trigger the conversion of bivalent chromosomes to dyads in *rec8-24A*/*rec8-24A* diploids.

### Nonphosphorylatable Rec8 Is Resistant to Separase

**Figure 2:**
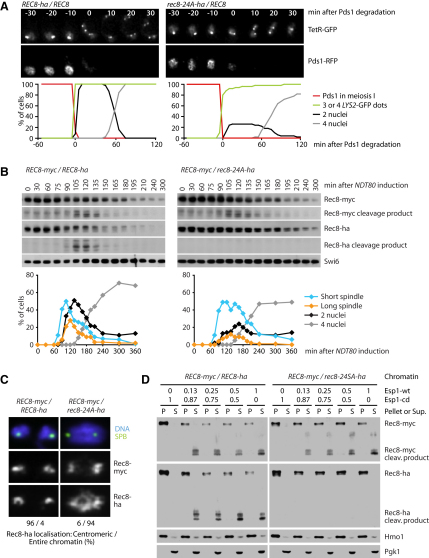
Rec8 Phosphorylation Is Required for Its Cleavage (A) Live imaging of meiosis in *REC8-ha/REC8* (Z12035) and *rec8-24A-ha/REC8* (Z12036) heterozygotes containing Pds1-RFP, TetR-GFP, and homozygous *LYS2-tetO*. Timing is relative to Pds1 degradation (t = 0) as in Figure 1D. See also Figure S2C. (B and C) *ndt80Δ* cells heterozygous for *REC8-myc/REC8-ha* (K15584) or *REC8-myc/rec8-24A-ha* (K15585) were released from arrest in prophase I by expressing *NDT80* from an estradiol-inducible promoter. Samples were collected at different times after release (t = 0). (B) Top: immunoblot detection of Rec8-myc, Rec8-ha, and Swi6 (loading control). Bottom: percentages of cells (n ≥ 100) with a short (blue) or a long (orange) spindle and two (black) or four (gray) nuclei. (C) Rec8-ha localization at centromeres or on the entire chromatin was quantified on chromosome spreads from anaphase I (n ≥ 50), where Rec8-myc is confined to the vicinity of SPBs (Tub4/γ-tubulin staining). (D) Cleavage of Rec8 in vitro. Chromatin isolated from the strains in (B), arrested in prophase I (but not released), was incubated with different mixtures of extracts from mitotic cells overproducing wild-type (wt, *esp1-1 P_GAL_-ESP1*, K8965) or catalytic-dead (cd, *esp1-1 P_GAL_-esp1-C1531A*, K8967) Esp1/separase. Reactions separated into chromatin pellet and supernatant were analyzed by anti-Myc and anti-Ha immunoblotting. Chromatin-associated Hmo1 and cytosolic Pgk1 served as loading controls.

If Rec8-24A blocked the meiosis I division due to its persistence on chromatin, it should prevent nuclear division in a dominant manner. To test this, we imaged Pds1-RFP and homozygous GFP-marked *LYS2* loci in *REC8-ha/REC8* or *rec8-24A-ha/REC8* heterozygotes (Figure 2A). Despite the frequent splitting of sister *LYS2* sequences upon Pds1-RFP destruction, the first meiotic division fails to take place in most (73%) *rec8-24A-ha/REC8* cells. Importantly, the deletion of *SPO11* restores this division (Figure S2C), from which we conclude that Rec8-24A is a dominant inhibitor of chiasmata resolution. Surprisingly, nuclear division in meiosis II occurs with only a small delay, suggesting that a critical amount of sister chromatid cohesion may be required to resist spindle forces effectively.

To investigate whether phosphorylation is necessary for Rec8's cleavage by separase, we used the C-terminal Myc and Ha tags to compare the abundance of Rec8 cleavage products in heterozygous *REC8-myc/REC8-ha* and *REC8-myc/rec8-24A-ha* cells (Figure 2B). To facilitate detection of the short-lived cleavage products, we synchronized our meiotic cultures by using a pachytene arrest/release protocol. After transfer to sporulation medium, cells arrest in pachytene due to a deletion of the *NDT80* gene. Cells are then released to synchronously progress through meiosis I by expressing *NDT80* from an estradiol-inducible promoter. In *REC8-myc/REC8-ha* cells, the full-length proteins of both Rec8 versions start to decline, and their cleavage products accumulate (transiently) 45 min after spindle formation (Figure 2B, left). Cleavage of wild-type Rec8-myc proceeds with similar kinetics in *REC8-myc/rec8-24A-ha* cells, but this is neither accompanied by a major decline in full-length Rec8-24A-ha protein nor by the appearance of Ha-tagged cleavage products, and 70% of the cells fail to undergo the first nuclear division (Figure 2B, right). Although Rec8-24A is not cleaved by separase, it does not hinder the activation of the protease. Next, we measured the association of Ha- and Myc-tagged proteins with chromatin from anaphase I cells (Figure 2C). Wild-type Rec8-ha and Rec8-myc colocalize and accumulate exclusively within pericentric chromatin surrounding each SPB. In contrast, in cells coexpressing Rec8-24A-ha and Rec8-myc, Ha-tagged protein colocalizes with the bulk of chromatin, and only Myc-tagged protein surrounds the SPBs. These data imply that Rec8-24A is neither cleaved nor removed from chromatin upon activation of separase in meiosis I. As a consequence, it inhibits meiosis I nuclear division in a dominant manner.

Finally, to demonstrate that nonphosphorylatable Rec8 is a poor substrate for separase in vitro, we incubated chromatin isolated from meiotic *REC8-myc/REC8-ha* and *REC8-myc/rec8-24A-ha* cells with extracts from mitotic cells that overproduce separase (Figure 2D). Separation of these reactions into an insoluble chromatin fraction and supernatant revealed that both Rec8-myc and Rec8-ha are cleaved by wild-type separase, but not by a “catalytic-dead” version. In the presence of active separase, full-length Rec8-myc and Rec8-ha disappear from the chromatin fraction while a cleavage product appears in the supernatant. Rec8-24A, in contrast, is poorly cleaved and remains in the chromatin pellet, even when wild-type Rec8-myc is readily cleaved by separase in the same extract. These data suggest that the cleavability of Rec8 depends on its phosphorylation status rather than on any meiosis-specific regulation of separase.

### Phosphomimetic Rec8 Mutants Cause Precocious Separation of Sister Centromeres

**Figure 3:**
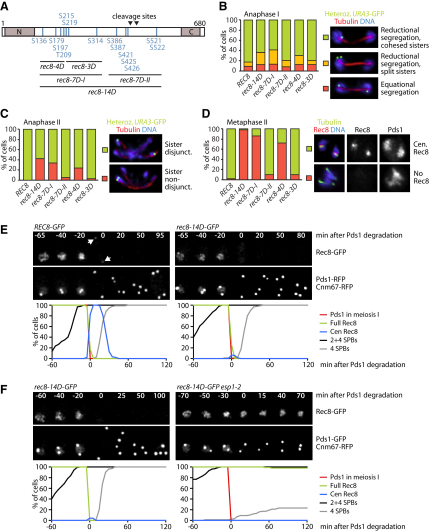
Phosphomimetic Rec8 Mutants Are Not Protected at Centromeres (A) Phosphomimetic Rec8 mutants generated by changing the indicated residues to aspartate. Regions conserved between α-kleisins are gray. (B–D) Immunofluorescence microscopy of meiotic *REC8-ha* (K15246), *rec8-14D-ha* (K15247), *rec8-7D-I-ha* (K16271), *rec8-7D-II-ha* (K16272), *rec8-4D-ha* (K16420), and *rec8-3D-ha* (K16421) cells containing Pds1-myc, TetR-GFP, and heterozygous *URA3-tetO*. (B) Quantification of the pattern of *URA3* sister segregation at anaphase I (n = 100). (C) Quantification of *URA3* sister nondisjunction at anaphase II (n = 100). (D) Quantification of the presence of centromeric Rec8 at metaphase II (n = 100). (E and F) Live imaging of Rec8-GFP, Cnm67-RFP at SPBs, and Pds1-RFP in meiosis. Timing is relative to Pds1 degradation (t = 0) as in Figure 1B. Arrows mark centromeric Rec8. (E) *REC8-GFP* (Z12781, from Figure 1B) and *rec8-14D-GFP* (Z12783) cells at 30°C. (F) *rec8-14D-GFP* (Z12783) and *rec8-14D-GFP esp1-2* (Z15642) cells at 34°C. See also Figure S3.

The finding that Rec8 phosphorylation promotes its cleavage suggests that cohesin's persistence at centromeres until meiosis II might be conferred by Rec8's selective dephosphorylation by PP2A at this location. If so, replacement of serines or threonines whose phosphorylation promotes cleavage by a phosphomimetic residue such as aspartate might confer phosphorylation-independent cleavage. PP2A should not protect the phosphomimetic form, which would be cleaved at centromeres at the same time as cleavage along chromosome arms, leading to precocious sister centromere separation and nondisjunction at meiosis II. To test this, we replaced with aspartate the 12 serines and threonines from our first round of phosphosite mapping plus 2 residues close to the separase cleavage sites, creating the *rec8-14D* allele. In addition, we created *rec8-D* mutants with different subsets of these substitutions (Figure 3A). To analyze sister chromatid cohesion, one copy of chromosome V was marked with GFP at the *URA3* locus, 35 kb from the centromere.

Due to monopolin activity, sister centromeres segregate to the same pole at anaphase I in 90% of wild-type cells, a phenomenon unaltered by any of the *rec8-D* mutations (Figure 3B). In contrast, *rec8-14D* and, to a lesser extent, *rec8-7D-I* and *rec8-4D* cause a noticeable increase in the frequency of anaphase I cells with separated sister *URA3* sequences (Figure 3B). *rec8-14D*, *rec8-7D-I*, and *rec8-4D* also cause large increases in the frequency of sister centromere nondisjunction at anaphase II (40%, 33%, and 24%, respectively; Figure 3C). We conclude that phosphomimetic substitutions within Rec8's N-terminal half cause the precocious separation of sister centromeres. To test whether this phenotype is due to the cleavage of centromeric cohesin at meiosis I, we used immunofluorescence microscopy to detect Rec8 in metaphase II cells. Rec8 is observed in the vicinity of SPBs in most wild-type cells (98%), but only rarely in *rec8-D* mutants with phosphomimicking substitutions in the N terminus (*rec8-14D*, 2%; *rec8-7D-I*, 14%; *rec8-4D*, 28%; Figure 3D).

We also analyzed the *rec8-14D* allele by using live imaging. Homozygous *rec8-14D-GFP* cells progress through meiosis with normal kinetics, as judged by the separation of RFP-marked SPBs and the degradation of Pds1-RFP (Figure S3). However, the Rec8-14D-GFP protein fails to persist at centromeres after the degradation of Pds1 in meiosis I (Figure 3E). Importantly, the disappearance of Rec8-14D-GFP at anaphase I is abolished by the *esp1-2* mutation, which inactivates separase at 34°C (Figure 3F). Rec8-14D is not, therefore, removed from chromosomes by a separase-independent mechanism (Yu and Koshland, 2005). We conclude that phosphomimetic substitutions cause Rec8 to be cleaved by separase at centromeres as well as along chromosome arms during meiosis I.

### Phosphomimetic Rec8 Restores the First Nuclear Division in Monopolin Mutants

**Figure 4:**
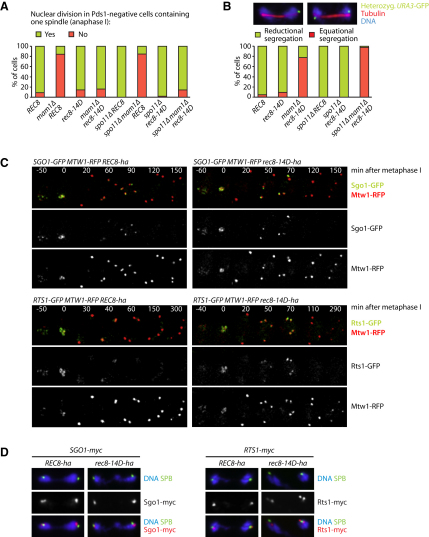
Phosphomimetic Rec8 Supports Sister Centromere Cohesion in Metaphase I and Does Not Affect the Localization of Sgo1-PP2A (A and B) Immunofluorescence microscopy of meiotic cells containing Pds1-myc; TetR-GFP; heterozygous *URA3-tetO*; and *REC8-ha* (K15444), *mam1Δ REC8-ha* (K15448), *rec8-14D-ha* (K15247), *mam1Δ rec8-14D-ha* (K15449), *spo11Δ REC8-ha* (K15446), *spo11Δ mam1Δ REC8-ha* (K15450), *spo11Δ rec8-14D-ha* (K15447), or *spo11Δ mam1Δ rec8-14D-ha* (K15451). (A) Quantification of nuclear division at anaphase I (Pds1-negative, one spindle; n = 100). (B) Quantification of reductional/equational *URA3* sister segregation in strains able to divide their nuclei in meiosis I (n = 100). (C) Live imaging of meiosis in *SGO1-GFP MTW1-RFP* cells with *REC8-ha* (Z14855) or *rec8-14D-ha* (Z14860) and *RTS1-GFP MTW1-RFP* cells with *REC8-ha* (Z15523) or *rec8-14D-ha* (Z15526). Time (min) is relative to entry into metaphase I, during which Mtw1 foci coalesce into a single cluster. (D) Chromosome spreads from *SGO1-myc* cells with *REC8-ha* (K15991) or *rec8-14D-ha* (K15992) and *RTS1-myc* cells with *REC8-ha* (K15993) or *rec8-14D-ha* (K15994) in anaphase I were stained for DNA, Tub4/γ-tubulin at SPBs, and Myc.

To address whether Rec8-14D is cleaved at centromeres at the same time as along chromosome arms at meiosis I, we tested whether the aspartate substitutions suppress the inability of *mam1Δ* cells, which lack monopolin, to undergo the first meiotic division. Sister kinetochores are pulled to opposite poles at meiosis I in *mam1Δ* cells but cannot disjoin due to the resistance of centromeric cohesin to separase activity. This results in an accumulation of Pds1-negative, mononucleate cells with a single bipolar spindle (Figure 4A). A failure to protect centromeric cohesin from separase, as occurs in *sgo1Δ* or *rts1Δ* mutants, or in cells that produce Scc1 in meiosis instead of Rec8, enables *mam1Δ* cells to divide their nuclei at meiosis I. *rec8-14D* has a similar effect (Figure 4A). Due to chiasmata, sister centromeres segregate to opposite poles in only 76% of cases in *rec8-14D mam1Δ* cells (Figure 4B). However, the elimination of recombination by deleting *SPO11* enables almost all *rec8-14D mam1Δ spo11Δ* cells to disjoin sister centromeres at meiosis I (Figure 4B). A corollary is that Rec8-14D is not simply defective in conferring sister centromere cohesion because if it were, efficient biorientation of sister centromeres would not be possible in *rec8-14D mam1Δ spo11Δ* triple mutants. Rec8-14D creates cohesion at centromeres, but it cannot persist after meiosis I separase activation.

### Phosphomimetic Rec8 Does Not Alter the Association of Sgo1 and PP2A with Centromeres

Substitution of serines and threonines by aspartate causes precocious cleavage of centromeric Rec8 either because it mimics the effect of phosphorylation, which is both necessary and sufficient to confer cleavability by separase, or because it somehow prevents the association of Sgo1 or PP2A with centromeres. If the latter were the case, PP2A's crucial substrate could be a protein other than Rec8. However, live imaging of Sgo1-GFP and the kinetochore protein Mtw1-RFP reveal similar levels of Sgo1 during metaphase I in *REC8* and *rec8-14D* cells (Figure 4C). Likewise, the *rec8-14D* allele has no detectable effect on the localization of Rts1-GFP at kinetochores. The levels of Sgo1 and Rts1 at kinetochores drop markedly as cells enter anaphase I, only to increase again at metaphase II. On chromosome spreads, however, both proteins can still be detected in the vicinity of SPBs during anaphase I (Figure 4D). We conclude that the precocious cleavage of centromeric Rec8-14D at meiosis I cannot be caused by the loss of Sgo1 or PP2A from centromeres. Instead, it must be due to PP2A's inability to prevent cleavage of Rec8-14D.

### The Protein Kinases Hrr25/CK1δ/ɛ, DDK, and Cdc5/PLK Bind to Rec8

**Figure 5:**
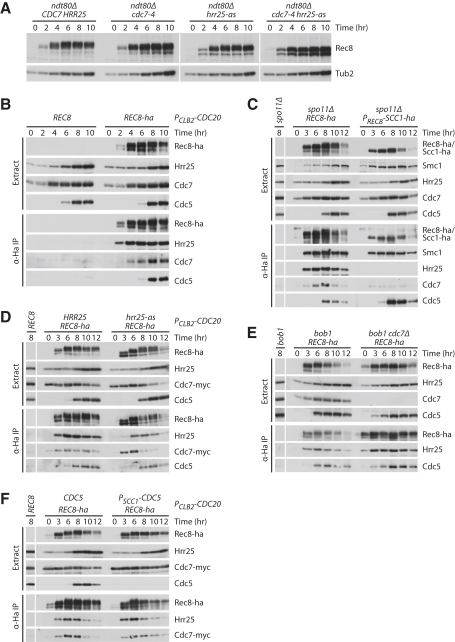
Interaction of Rec8 with the Protein Kinases Hrr25, DDK, and Cdc5 (A) Immunoblot detection of Rec8 in extracts from *ndt80Δ* cells with *CDC7 HRR25* (Z16490), *cdc7-4* (Z16491), *hrr25-as* (Z16492), or *cdc7-4 hrr25-as* (Z16492) entering meiosis (31°C, 1NM-PP1). (B–F) Immunoblot analysis of anti-Ha immunoprecipitates from extracts of meiotic *REC8* (control) and *REC8-ha* strains. (B) *P_CLB2_-CDC20* cells with *REC8* (Z5620) or *REC8-ha* (Z7532). (C) *spo11Δ* cells with *REC8* (Z7271), *REC8-ha* (Z8225), or *P_REC8_-SCC1-ha* (Z8444). (D) *P_CLB2_-CDC20* cells with *REC8* (Z10271), *REC8-ha* (Z10266), or *REC8-ha hrr25-as* (Z10274), treated with 1NM-PP1. (E) *bob1* cells with *REC8* (Z9053), *REC8-ha* (Z9052), or *REC8-ha cdc7Δ* (Z9341). (F) *P_CLB2_-CDC20* cells with *REC8* (Z8535), *REC8-ha* (Z8536), or *REC8-ha P_SCC1_-CDC5* (Z9320).

What kinases are responsible for the Rec8 phosphorylation necessary for its cleavage? In mitotic cells, Cdc5/PLK promotes cohesin cleavage by phosphorylating Rec8's mitotic counterpart Scc1 (Alexandru et al., 2001; Hornig and Uhlmann, 2004). Because Cdc5 also regulates Rec8 phosphorylation, it has been assumed, but never demonstrated, that Cdc5 also promotes cohesin cleavage in meiosis (Brar et al., 2006; Clyne et al., 2003; Lee and Amon, 2003). However, certain mutations allow for the efficient cleavage of Rec8 prior to Cdc5's appearance. For example, *mnd2Δ ndt80Δ* cells activate the meiosis-specific APC/C-Ama1 prematurely due to the absence of the APC/C inhibitor Mnd2. This causes separase activation and Rec8 cleavage in the absence of Cdc5, whose accumulation depends on Ndt80 (Oelschlaegel et al., 2005; Penkner et al., 2005). Phosphorylation of Rec8 by Cdc5 is not, therefore, obligatory for cleavage, and other protein kinases must be involved. Good candidates are Hrr25/CK1δ/ɛ and DDK because these kinases are required for the normal phosphorylation of Rec8 not only in metaphase I (Matos et al., 2008; Petronczki et al., 2006) but also during prophase I (Figure 5A).

Rec8 might be expected to copurify with its kinases. Thus, we measured the abundance of the three kinases in immunoprecipitates of Rec8-ha prepared at different times after the induction of meiosis. Hrr25 and Cdc7 coprecipitate with Rec8 from early and mid-prophase I onward, respectively. Cdc5 copurifies with Rec8 only from metaphase I forward (Figure 5B). Interestingly, neither Hrr25 nor Cdc7 associate with Scc1 when expressed during meiosis instead of Rec8. In contrast, Cdc5 associates with both kleisin subunits and, if anything, preferentially with Scc1 (Figure 5C). Importantly, association of each kinase is independent of the activity of the others. Thus, neither inhibition of the analog-sensitive Hrr25-as kinase with 1NM-PP1 (Figure 5D) nor deletion of *CDC7* (only possible in *bob1* mutants) (Figure 5E) nor depletion of Cdc5 (Figure 5F) has any effect on Rec8's association with the remaining two kinases. There is, therefore, no evidence that the activity of earlier kinases “primes” Rec8 to associate with later ones.

### DDK and Hrr25 Are Required for Rec8 Cleavage in *mnd2Δ* Mutants

**Figure 6:**
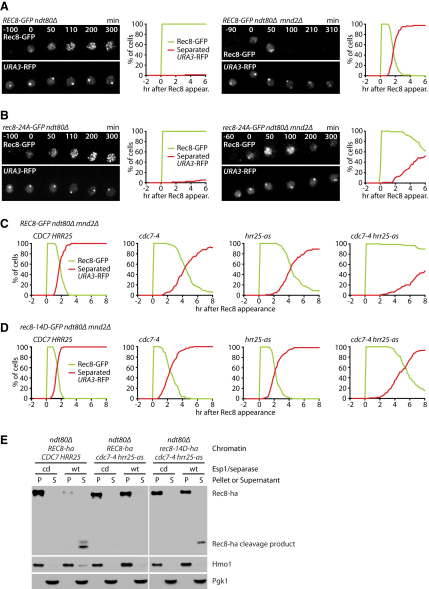
Hrr25 and DDK Promote Cohesin Destruction upon APC/C Activation in Prophase I (A–D) Live imaging of Rec8-GFP and TetR-RFP in heterozygous *URA3-tetO* strains entering meiosis at 31°C. Panels: time-lapse series with time (min) after Rec8 appearance. Graphs: the presence of nuclear Rec8-GFP (green) and separated *URA3*-RFP sister sequences (red) was quantified every 10 min in 100 cells, in which Rec8 appearance was set to t = 0. (A) *REC8-GFP ndt80Δ* cells with *MND2* (Z14429) or *mnd2Δ* (Z14432). (B) *rec8-24A-GFP ndt80Δ* cells with *MND2* (Z14489) or *mnd2Δ* (Z14492). (C) *REC8-GFP ndt80Δ mnd2Δ* cells with *CDC7 HRR25* (Z15180), *cdc7-4* (Z15434), *hrr25-as* (Z15436), or *hrr25-as cdc7-4* (Z15215), treated with 1NM-PP1. (D) *rec8-14D-GFP ndt80Δ mnd2Δ* cells with *CDC7 HRR25* (Z15181), *cdc7-4* (Z15433), *hrr25-as* (Z15435), or *hrr25-as cdc7-4* (Z15182), treated with 1NM-PP1. (E) Cleavage of Rec8 in vitro. *ndt80Δ REC8-ha* cells (K17066) and *ndt80Δ cdc7-4 hrr25-as* cells with *REC8-ha* (K17068) or *rec8-14D-ha* (K17069) were arrested in prophase I (31°C, 1NM-PP1). Chromatin was isolated and incubated with inactive (cd) or active (wt) Esp1/separase as in Figure 2D. Reactions separated into pellet and supernatant were analyzed by anti-Ha immunoblotting. See also Figure S4.

To investigate whether Hrr25 and/or DDK promotes cohesin cleavage during prophase I, we filmed Rec8-GFP and *URA3* sister sequences marked with RFP in *ndt80Δ* cells in the presence or absence of Mnd2. In *ndt80Δ* cells containing Mnd2, Rec8-GFP persists on chromosomes and sisters remain tightly associated (Figure 6A, left). In the absence of Mnd2, Rec8-GFP disappears from chromosomes ∼2 hr after its accumulation, and this is accompanied by sister separation (Figure 6A, right). Crucially, both Rec8's disappearance and sister separation are greatly delayed in *ndt80Δ mnd2Δ* cells homozygous for *rec8-24A-GFP* (Figure 6B, right), confirming that Rec8 cleavage is promoted by phosphorylation under these conditions. The levels of Rec8-24A-GFP do eventually decline, possibly due to residual phosphorylation and the persistence of separase activity.

To reduce DDK activity, *ndt80Δ mnd2Δ* cells containing the temperature-sensitive *cdc7-4* allele were shifted to 31°C. Under these conditions, DNA replication proceeds normally, whereas recombination and monopolar attachment are defective (Matos et al., 2008). Reduced DDK activity causes a modest delay in the removal of Rec8 (Figure 6C, second panel). Rec8 removal is similarly delayed in *bob1* mutants lacking the entire *CDC7* gene (Figure S4A). To inhibit Hrr25, we treated *ndt80Δ mnd2Δ* cells containing *hrr25-as* with 1NM-PP1. This also causes a moderate delay in Rec8's disappearance (Figure 6C, third panel). Remarkably, simultaneous inhibition of both kinases has a far greater effect, blocking Rec8's disappearance for several hours (Figure 6C, fourth panel). We also detected Rec8 by immunoblotting in protein extracts from *ndt80Δ mnd2Δ* cells, whose *UBR1* gene had been deleted to stabilize Rec8's C-terminal cleavage product (Buonomo et al., 2000) (Figure S4B). Consistent with our imaging results, full-length Rec8 remains at high levels, and production of the cleavage product is diminished only when both kinases are inhibited or phosphosites are mutated to alanine. These data suggest that DDK and Hrr25 both promote the cleavage-dependent removal of Rec8 from chromosomes, at least when separase is activated prematurely during prophase I in *ndt80Δ mnd2Δ* mutants. Accordingly, Rec8 on chromatin isolated from prophase I-arrested *cdc7-4 hrr25-as* double mutants is a very poor substrate for separase in vitro (Figure 6E).

If the kinases exerted their effect by phosphorylating Rec8, then the delayed removal of Rec8 should be abrogated by replacing *REC8* with the *rec8-14D* allele. This was partly the case. The Rec8-14D-GFP protein disappears more rapidly than Rec8-GFP when Cdc7 and Hrr25 are inhibited, either separately or together, although not as rapidly as in cells with active Cdc7 and Hrr25 kinases (Figure 6D). The aspartate substitutions also increase the cleavage of Rec8 on chromatin from *cdc7-4 hrr25-as* cells by separase in vitro (Figure 6E). Because Cdc5 is not expressed in *ndt80Δ* mutants (Clyne et al., 2003), it cannot have any role in facilitating Rec8 cleavage in these cells. Even when expressed ectopically during prophase I from the *DMC1* promoter, Cdc5 fails to accelerate Rec8's removal in *ndt80Δ mnd2Δ* cells with or without Hrr25 activity (Figures S4C and S4D).

### DDK and Hrr25, but Not Cdc5, Are Required for Rec8 Cleavage at Anaphase I

**Figure 7:**
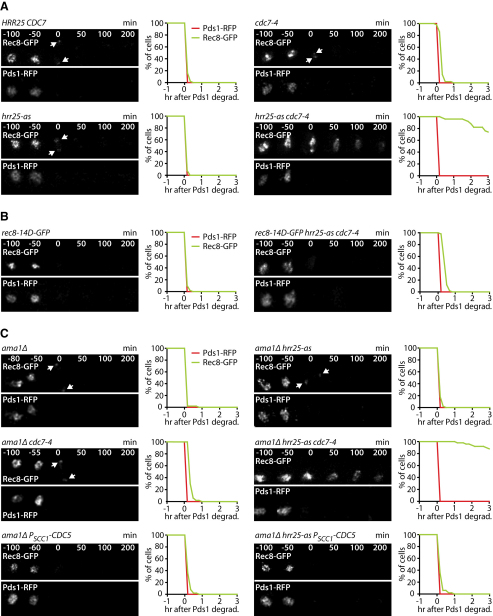
Rec8 Phosphorylation by Hrr25 and DDK, but Not Cdc5, Is Required for Cleavage at Anaphase I (A–C) Live imaging of Rec8-GFP and Pds1-RFP in meiosis (31°C, 1NM-PP1). Panels: time-lapse series with time (min) after Pds1 degradation. Arrows mark centromeric Rec8. Graphs: the presence of nuclear Rec8-GFP (green) and Pds1-RFP (meiosis I, red) was quantified every 10 min in 50 cells, in which Pds1 degradation was set to t = 0. (A) *REC8-GFP* cells with *HRR25 CDC7* (Z15135), *cdc7-4* (Z15055), *hrr25-as* (Z15138), or *hrr25-as cdc7-4* (Z15058). (B) *rec8-14D-GFP* (Z15438) and *rec8-14D-GFP hrr25-as cdc7-4* (Z15439) cells. (C) *REC8-GFP ama1Δ* cells with *HRR25 CDC7* (Z16346), *hrr25-as* (Z15615), *cdc7-4* (Z15703), *hrr25-as cdc7-4* (Z15704), *P_SCC1_-CDC5* (Z15612), or *hrr25-as P_SCC1_-CDC5* (Z15614). See also Figures S5 and S6.

Are DDK and Hrr25 also important for Rec8's removal from chromosome arms at the onset of anaphase I? To address this, we filmed cells containing Rec8-GFP and Pds1-RFP. Inactivation of either Cdc7 or Hrr25 alone has little effect on the kinetics of Rec8's disappearance (Figure 7A, top right and bottom left). In contrast, simultaneous inhibition of both kinases causes Rec8 to persist on chromosome arms for several hours after Pds1 destruction (Figure 7A, bottom right), even in cells lacking Sgo1 (Figure S5A). Due to bioriented sister centromeres, *cdc7-4* and *hrr25-as* single mutants fail to undergo the first meiotic division unless protection of centromeric cohesin from separase is abrogated (Matos et al., 2008; Petronczki et al., 2006). In contrast, most (74%) *cdc7-4 hrr25-as* double mutant cells fail to divide their nuclei at meiosis I even in the absence of Sgo1, indicating a strong delay in Rec8 cleavage (Figure S5B). Importantly, the Rec8-14D protein disappears upon Pds1 degradation in the double kinase mutant cells (Figure 7B). This implies that the persistence of wild-type Rec8 is due to a reduction in its phosphorylation. Our data suggest that phosphorylation of Rec8 by either DDK or Hrr25 promotes cohesin cleavage on chromosome arms. In the absence of both kinases, Rec8 is much more resistant to separase. Cells that produce Scc1 during meiosis instead of Rec8 undergo nuclear division after inhibition of both DDK and Hrr25, demonstrating that Rec8's dependence on these kinases for its cleavage is not a general property of α-kleisins (Figure S5C). Hrr25's activity in monopolar attachment depends on its binding to the Mam1 subunit of monopolin, which is dispensable for Rec8 cleavage. To confirm that Hrr25 promotes Rec8 cleavage independently of monopolin, we analyzed *hrr25-zo* strains, in which Hrr25 cannot bind to Mam1 (Petronczki et al., 2006). As expected, Rec8 disappears with normal kinetics in both *hrr25-zo* single and *hrr25-zo cdc7-4* double mutants (Figure S5D).

**Figure 8:**
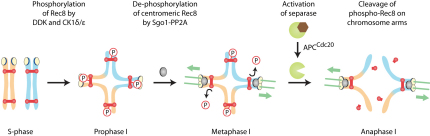
Model for the Control of Rec8 Cleavage by Rec8 Kinases, Sgo1-PP2A, and Separase CK1δ/ɛ and DDK phosphorylate Rec8 along chromosomes, and this phosphorylation is prevented at centromeres by Sgo1-PP2A. At the onset of anaphase I, separase cleaves phosphorylated Rec8 on chromosome arms, leading to chiasmata resolution. Unphosphorylated, centromeric Rec8 is resistant to separase and persists to support the biorientation of sister chromatids in meiosis II. See also Figure S7.

Our results imply that Cdc5 cannot alone promote Rec8 cleavage at the onset of anaphase I. This does not exclude an auxiliary role. The fact that Cdc5 is required for Pds1 degradation in meiosis I (Clyne et al., 2003; Lee and Amon, 2003) has so far precluded any rigorous analysis of its function in cohesin cleavage. However, we have discovered that elimination of the meiosis-specific APC/C activator Ama1 renders Pds1 degradation independent of Cdc5 (Figure S6A). This enabled us to analyze the effects of Cdc5 depletion on processes that normally depend on Pds1 degradation. In *ama1Δ* mutants, Rec8 is cleaved with similar kinetics as in wild-type cells (Figure 7C, top left). Inactivation of either Hrr25 or Cdc7 has little if any effect, and simultaneous inhibition of both kinases blocks Rec8 cleavage, as observed in Ama1-containing cells (Figure 7C, top right, middle left, and middle right, respectively). Interestingly, Cdc5 depletion does not retard Rec8's removal from chromosomes upon Pds1 degradation in *ama1Δ* cells (Figure 7C, bottom left), even when Hrr25 is also inactivated (Figure 7C, bottom right). This experiment also revealed that little or no Rec8 persists at centromeres after Pds1 destruction in *ama1Δ* cells lacking Cdc5 (Figure 7C, bottom left). We confirmed this surprising observation by detecting Rec8 on chromosome spreads (Figure S6B). These data suggest that Cdc5 has no direct role in promoting Rec8 cleavage. Indeed, it seems to play a quite different role, namely, helping to protect centromeric Rec8 from separase. In conclusion, we propose that cohesin cleavage at meiosis I requires the phosphorylation of Rec8 by DDK and Hrr25. By removing these phosphate groups, PP2A bound to Sgo1 protects centromeric cohesin from separase (Figure 8). Because phosphomimicking mutations in Rec8's N terminus stimulate cleavage several hundred residues away, separase might recognize phosphorylated Rec8 with its large noncatalytic domain rather than its C-terminal protease domain (Figures S7A and S7B).

## Discussion

Sister chromatid cohesion established during premeiotic DNA replication is capable of mediating two rounds of chromosome segregation because it is destroyed in a stepwise manner. Separase activation at meiosis I resolves bivalent chromosomes into dyads by cleavage of cohesin's meiosis-specific kleisin subunit Rec8 on chromosome arms. Cohesin at centromeres persists, however, and holds dyads together until their resolution into single chromatids by a second burst of separase activity in meiosis II. The finding that protection of centromeric cohesin from separase requires binding of PP2A to kinetochore proteins known as shugoshins suggests that phosphorylation either of separase or its target might be essential for cohesin cleavage in meiosis.

Cohesin's kleisin subunit has been considered the prime suspect, largely because cleavage in mitosis is facilitated by the phosphorylation of Scc1 by Cdc5/PLK (Alexandru et al., 2001; Hauf et al., 2005; Hornig and Uhlmann, 2004). The prevailing model is that PP2A recruited to centromeres by Sgo1 protects Rec8 from separase by removing phosphate groups produced by Cdc5, which are essential for its cleavage at meiosis I. Despite a clear role for Cdc5 in Rec8 phosphorylation (Clyne et al., 2003; Lee and Amon, 2003), the model has two major flaws. First, substitution by alanine of multiple serines and threonines within Rec8 thought to be phosphorylated by Cdc5 during meiosis I was not found to have any major effect on the kinetics of Rec8 cleavage (Brar et al., 2006). Second, if Cdc5 promoted kleisin cleavage during meiosis and PP2A reversed this process at centromeres, why is Scc1 not protected from separase at centromeres when expressed instead of Rec8 (Tóth et al., 2000)?

Our conclusion that the phosphorylation of Rec8 is essential for its cleavage is based on three lines of evidence. First, alanine substitution of 24 serine/threonine residues phosphorylated in vivo blocks Rec8 cleavage and hinders chiasmata resolution in a dominant manner despite normal activation of separase. Second, two different kinases known to phosphorylate Rec8, Hrr25/CK1δ/ɛ and DDK, are required for its cleavage, but not for separase activation. Third, phosphomimetic substitution of phosphorylated residues by aspartate enables separase to cleave Rec8 on chromosome arms and at centromeres, even when both kinases are inhibited. We propose that Hrr25- and DDK-dependent phosphorylation of Rec8 promotes cohesin cleavage in meiosis I, whereas dephosphorylation of Rec8 by PP2A bound to Sgo1 protects it from separase at centromeres (Figure 8). Our findings help to explain how a single round of sister chromatid cohesion supports two rounds of chromosome segregation. We also show that Cdc5 has little, if any, role in promoting Rec8 cleavage, which is consistent with the finding that mutations of Cdc5-dependent phosphosites have little effect on the kinetics of Rec8 cleavage, and that premature APC/C activation in prophase I causes efficient Rec8 cleavage in the absence of Cdc5 (Brar et al., 2006; Oelschlaegel et al., 2005; Penkner et al., 2005). Also in fission yeast, resolution of bivalents is promoted by CK1δ/ɛ- but not PLK-dependent phosphorylation of Rec8 (Y. Watanabe, personal communication), demonstrating that this process is evolutionarily conserved.

### Control of Rec8 Cleavage by Multiple Phosphorylation Sites

Although mutation of 24 serine/threonine residues is necessary to prevent Rec8 cleavage in meiosis I, it is conceivable that cleavage of the wild-type kleisin is promoted by a smaller number of phosphosites. Aspartate is probably a less than perfect substitute for phosphoserine and phosphothreonine. Nevertheless, substitution of only four amino acids induces precocious loss of sister centromere cohesion in a large fraction of meiotic cells. The simplest explanation for this finding is that phosphorylation of at least four residues is sufficient to transform Rec8 into an effective separase substrate, but that there is considerable flexibility concerning their identity and location. When residues preferred as substrates by DDK and Hrr25 are mutated, then phosphorylation of others can also promote cleavage, which ultimately only fails when most (up to 24) potential phosphorylation sites have been mutated.

One value of multisite phosphorylation is that it can facilitate ultrasensitive “switch-like” responses, such as occurs in the degradation of the CDK inhibitor Sic1 at the G1/S transition (Nash et al., 2001). At the onset of anaphase I, separase must cleave Rec8 efficiently on chromosome arms; a failure to cleave only a few molecules could hinder the resolution of chiasmata. Indeed, this might be a reason for the involvement of two different protein kinases when only one is strictly necessary. Meanwhile, however, centromeric Rec8 must be protected from separase activity to prevent precocious separation of sister centromeres. The requirement for multisite phosphorylation would facilitate a bistable “switch-like” process whereby modest changes in the phosphatase/kinase activity ratio between chromosome arms and centromeres results in only two states of Rec8—either a highly phosphorylated, cleavable form at chromosome arms or a poorly phosphorylated, noncleavable form at centromeres. If only one phosphorylation site were used to differentiate arm from centromeric Rec8, efficient cleavage of Rec8 on chromosome arms might require extremely high kinase activities to ensure the phosphorylation of all of Rec8. Whereas multisite phosphorylation of Sic1 provides a temporal switch in CDK activation, multisite phosphorylation of Rec8 might work as a spatial switch, in which one pool of chromosome-bound Rec8 is discriminated from the other.

How does separase recognize phosphorylated Rec8? It is remarkable that aspartates, and by implication phosphate groups, in the N terminus of Rec8 are much more potent in promoting cleavage than aspartates close to the cleavage sites. We speculate, therefore, that phosphate groups stimulate cleavage by interacting with a part of separase that is distant from its catalytic center. Separase's C-terminal protease domain is preceded by a large noncatalytic domain that was proposed to contain multiple copies of a motif related to armadillo (ARM) repeats (Jäger et al., 2004). Our sequence alignments favor, however, the idea that this domain consists of tetratricopeptide (TPR) repeats (Figure S7A). TPR and also ARM repeats fold into flexible, superhelical structures that in certain cases are known to possess a long positively charged groove, which recognizes binding partners through multiple interactions of low affinity and variable position (Das et al., 1998; Huber et al., 1997). Indeed, modeling of separase's TPR repeats reveals an extended positively charged surface along the TPR superhelix (Figure S7B). We propose that the TPR repeats of separase bind to phosphate groups on Rec8 and thereby increase the concentration of the protease domain in the vicinity of Rec8's cleavage sites.

### Protection and Deprotection of Centromeric Cohesin

Protection of centromeric cohesin from separase in meiosis I depends on Sgo1, which recruits to kinetochores a PP2A phosphatase containing the B′ subunit Rts1. Our finding that separase removes a phosphomimetic version of Rec8 from centromeres at meiosis I implies that Rec8 and not separase is PP2A's substrate. We presume that PP2A dephosophorylates Rec8 itself, but we cannot formally exclude the possibility that PP2A protects centromeric cohesin by inhibiting Hrr25 and DDK. However, such an inhibition would require a mechanism that does not interfere with the functions of these kinases in monopolar attachment of sister kinetochores at meiosis I.

How does centromeric cohesin spared from separase in meiosis I become susceptible to the protease in meiosis II? Live imaging revealed that the concentration of Sgo1 and Rts1 at kinetochores decreases markedly upon the onset of anaphase I, but recovers as cells enter metaphase II. These data are consistent with the detection of yeast and animal shugoshins in the vicinity of kinetochores during meiosis II as well as during meiosis I (Gómez et al., 2007; Katis et al., 2004a; Kerrebrock et al., 1995; Lee et al., 2008; Marston et al., 2004). Shugoshin's recruitment of PP2A to kinetochores appears to be necessary for the spindle assembly checkpoint during mitosis (Indjeian et al., 2005; Xu et al., 2009), and this may be the reason why both proteins are present at meiosis II, which resembles mitosis. Shugoshin-PP2A complexes must therefore lose their ability to protect Rec8 from separase as cells enter meiosis II. Possibilities include the regulation of PP2A's activity by posttranslational modifications and subtle changes in the localization of Sgo1-PP2A, which are below the resolution of our microscopy system. It may be pertinent in this regard that the protection of centromeric cohesin appears to require Cdc5 (this work) as well as Spo13 (Katis et al., 2004b; Klein et al., 1999; Lee et al., 2004), a meiosis-specific protein that binds to Cdc5 (Matos et al., 2008) and disappears after meiosis I. Deprotection might involve the destruction of Spo13-Cdc5 complexes after meiosis I.

### Regulation of Rec8 Cleavage by Hrr25/CK1δ/ɛ and DDK

In addition to initiating meiotic DNA replication, DDK promotes DNA double-strand break formation, the first step of recombination, and the recruitment of the monopolin complex to kinetochores (Matos et al., 2008). Hrr25 is a subunit of monopolin (Petronczki et al., 2006) and is thought to mediate monopolar attachment through phosphorylation of as yet unknown substrates at kinetochores in meiosis I. Thus, the conversion of bivalent chromosomes to dyads at meiosis I is promoted by the very same kinases that have previously promoted their duplication, linkage through recombination, and mono-orientation of sister centromeres. It is remarkable that DDK and Hrr25 bind to and phosphorylate Rec8 already in S phase and mid-prophase, respectively. As a consequence, Rec8 is susceptible to separase already during prophase I. Consistent with this, premature degradation of Pds1 in prophase I in *mnd2Δ* mutants causes precocious Rec8 cleavage that is promoted by DDK and Hrr25 activity. This means that inhibition of APC/C-dependent securin degradation is the only mechanism that guards meiotic yeast cells against premature cohesin destruction. This might be relevant to human oocytes that arrest in prophase I for several decades and suffer from age-dependent chromosome nondisjunction that might be caused by a gradual loss of cohesin from chromosome arms.

## Experimental Procedures

### Yeast Strains

We used diploid *Saccharomyces cerevisiae* SK1 strains with the genotypes listed in Table S2. To create *rec8* mutants and control strains, plasmids carrying wild-type *REC8* or phosphosite mutants tagged with TAP, Ha, Myc, or GFP were integrated into the promoter of the *rec8Δ* locus (Buonomo et al., 2000). Tagged alleles of other genes have been described (Matos et al., 2008; Riedel et al., 2006).

### Induction of Meiosis

To induce meiosis, cells were transferred to sporulation medium (SPM) at 30°C as described (Petronczki et al., 2006). *cdc7-4* strains were transferred to SPM at 25°C and shifted to 31°C after 1 hr. Hrr25-as was inhibited with 1NM-PP1 (5 μM), added 0.5 and 3 hr after transfer to SPM. For pachytene arrest/release experiments, *ndt80Δ P_GAL1_-NDT80 P_GPD1_-GAL4-ER* cells were induced to enter meiosis for 7 hr and released into meiosis I with β-estradiol (1 μM) (Matos et al., 2008).

### Analysis of Proteins

Rec8-TAP was purified as described (Riedel et al., 2006), except that the lysis buffer contained phosphatase inhibitors (20 mM Na-pyrophosphate, 30 mM NaF, 60 mM β-glycerophosphate, 2 mM orthovanadate). Protein samples digested with trypsin, chymotrypsin, or subtilisin were separated by nano-HPLC and applied to a LTQ mass spectrometer. Immunoprecipitation, analysis of protein levels in TCA extracts and immunoblot detection of proteins was performed as described (Matos et al., 2008). Rec8-ha was cleaved in vitro as described (Buonomo et al., 2000; Uhlmann et al., 1999), except chromatin was isolated from *ndt80Δ* cells after 7 hr in SPM.

### Microscopy

For live imaging, meiotic cultures were transferred to a DeltaVision RT system and imaged every 5 or 10 min for 10–14 hr as described (Matos et al., 2008). Meiotic events were quantified in 50 or 100 individual cells, in which Pds1 degradation or Rec8 appearance was set to t = 0. Fixed cells and chromosome spreads were stained for immunofluorescence microscopy as described (Petronczki et al., 2006).

## Supplemental Information

Document S1. Seven Figures and Two Tables

## References

[bib1] Alexandru G., Uhlmann F., Mechtler K., Poupart M.A., Nasmyth K. (2001). Phosphorylation of the cohesin subunit Scc1 by Polo/Cdc5 kinase regulates sister chromatid separation in yeast. Cell.

[bib2] Brar G.A., Kiburz B.M., Zhang Y., Kim J.E., White F., Amon A. (2006). Rec8 phosphorylation and recombination promote the step-wise loss of cohesins in meiosis. Nature.

[bib3] Buonomo S.B., Clyne R.K., Fuchs J., Loidl J., Uhlmann F., Nasmyth K. (2000). Disjunction of homologous chromosomes in meiosis I depends on proteolytic cleavage of the meiotic cohesin Rec8 by separin. Cell.

[bib4] Clyne R.K., Katis V.L., Jessop L., Benjamin K.R., Herskowitz I., Lichten M., Nasmyth K. (2003). Polo-like kinase Cdc5 promotes chiasmata formation and cosegregation of sister centromeres at meiosis I. Nat. Cell Biol..

[bib5] Das A.K., Cohen P.W., Barford D. (1998). The structure of the tetratricopeptide repeats of protein phosphatase 5: implications for TPR-mediated protein-protein interactions. EMBO J..

[bib6] Gómez R., Valdeolmillos A., Parra M.T., Viera A., Carreiro C., Roncal F., Rufas J.S., Barbero J.L., Suja J.A. (2007). Mammalian SGO2 appears at the inner centromere domain and redistributes depending on tension across centromeres during meiosis II and mitosis. EMBO Rep..

[bib7] Hauf S., Roitinger E., Koch B., Dittrich C.M., Mechtler K., Peters J.M. (2005). Dissociation of cohesin from chromosome arms and loss of arm cohesion during early mitosis depends on phosphorylation of SA2. PLoS Biol..

[bib8] Hornig N.C., Uhlmann F. (2004). Preferential cleavage of chromatin-bound cohesin after targeted phosphorylation by Polo-like kinase. EMBO J..

[bib9] Huber A.H., Nelson W.J., Weis W.I. (1997). Three-dimensional structure of the armadillo repeat region of β-catenin. Cell.

[bib10] Indjeian V.B., Stern B.M., Murray A.W. (2005). The centromeric protein Sgo1 is required to sense lack of tension on mitotic chromosomes. Science.

[bib11] Jäger H., Herzig B., Herzig A., Sticht H., Lehner C.F., Heidmann S. (2004). Structure predictions and interaction studies indicate homology of separase N-terminal regulatory domains and *Drosophila* THR. Cell Cycle.

[bib12] Katis V.L., Galova M., Rabitsch K.P., Gregan J., Nasmyth K. (2004). Maintenance of cohesin at centromeres after meiosis I in budding yeast requires a kinetochore-associated protein related to MEI-S332. Curr. Biol..

[bib13] Katis V.L., Matos J., Mori S., Shirahige K., Zachariae W., Nasmyth K. (2004). Spo13 facilitates monopolin recruitment to kinetochores and regulates maintenance of centromeric cohesion during yeast meiosis. Curr. Biol..

[bib14] Kerrebrock A.W., Moore D.P., Wu J.S., Orr-Weaver T.L. (1995). Mei-S332, a *Drosophila* protein required for sister-chromatid cohesion, can localize to meiotic centromere regions. Cell.

[bib15] Kitajima T.S., Miyazaki Y., Yamamoto M., Watanabe Y. (2003). Rec8 cleavage by separase is required for meiotic nuclear divisions in fission yeast. EMBO J..

[bib16] Kitajima T.S., Kawashima S.A., Watanabe Y. (2004). The conserved kinetochore protein shugoshin protects centromeric cohesion during meiosis. Nature.

[bib17] Kitajima T.S., Sakuno T., Ishiguro K., Iemura S., Natsume T., Kawashima S.A., Watanabe Y. (2006). Shugoshin collaborates with protein phosphatase 2A to protect cohesin. Nature.

[bib18] Klein F., Mahr P., Galova M., Buonomo S.B., Michaelis C., Nairz K., Nasmyth K. (1999). A central role for cohesins in sister chromatid cohesion, formation of axial elements, and recombination during yeast meiosis. Cell.

[bib19] Kudo N.R., Wassmann K., Anger M., Schuh M., Wirth K.G., Xu H., Helmhart W., Kudo H., McKay M., Maro B. (2006). Resolution of chiasmata in oocytes requires separase-mediated proteolysis. Cell.

[bib20] Lee B.H., Amon A. (2003). Role of Polo-like kinase CDC5 in programming meiosis I chromosome segregation. Science.

[bib21] Lee B.H., Kiburz B.M., Amon A. (2004). Spo13 maintains centromeric cohesion and kinetochore coorientation during meiosis I. Curr. Biol..

[bib22] Lee J., Kitajima T.S., Tanno Y., Yoshida K., Morita T., Miyano T., Miyake M., Watanabe Y. (2008). Unified mode of centromeric protection by shugoshin in mammalian oocytes and somatic cells. Nat. Cell Biol..

[bib23] Marston A.L., Tham W.H., Shah H., Amon A. (2004). A genome-wide screen identifies genes required for centromeric cohesion. Science.

[bib24] Matos J., Lipp J.J., Bogdanova A., Guillot S., Okaz E., Junqueira M., Shevchenko A., Zachariae W. (2008). Dbf4-dependent Cdc7 kinase links DNA replication to the segregation of homologous chromosomes in meiosis I. Cell.

[bib25] Nash P., Tang X., Orlicky S., Chen Q., Gertler F.B., Mendenhall M.D., Sicheri F., Pawson T., Tyers M. (2001). Multisite phosphorylation of a CDK inhibitor sets a threshold for the onset of DNA replication. Nature.

[bib26] Nasmyth K., Haering C.H. (2005). The structure and function of SMC and kleisin complexes. Annu. Rev. Biochem..

[bib27] Oelschlaegel T., Schwickart M., Matos J., Bogdanova A., Camasses A., Havlis J., Shevchenko A., Zachariae W. (2005). The yeast APC/C subunit Mnd2 prevents premature sister chromatid separation triggered by the meiosis-specific APC/C-Ama1. Cell.

[bib28] Penkner A.M., Prinz S., Ferscha S., Klein F. (2005). Mnd2, an essential antagonist of the anaphase-promoting complex during meiotic prophase. Cell.

[bib29] Petronczki M., Siomos M.F., Nasmyth K. (2003). Un ménage à quatre: the molecular biology of chromosome segregation in meiosis. Cell.

[bib30] Petronczki M., Matos J., Mori S., Gregan J., Bogdanova A., Schwickart M., Mechtler K., Shirahige K., Zachariae W., Nasmyth K. (2006). Monopolar attachment of sister kinetochores at meiosis I requires casein kinase 1. Cell.

[bib31] Rabitsch K.P., Gregan J., Schleiffer A., Javerzat J.P., Eisenhaber F., Nasmyth K. (2004). Two fission yeast homologs of *Drosophila* Mei-S332 are required for chromosome segregation during meiosis I and II. Curr. Biol..

[bib32] Riedel C.G., Katis V.L., Katou Y., Mori S., Itoh T., Helmhart W., Gálová M., Petronczki M., Gregan J., Cetin B. (2006). Protein phosphatase 2A protects centromeric sister chromatid cohesion during meiosis I. Nature.

[bib33] Tóth A., Rabitsch K.P., Gálová M., Schleiffer A., Buonomo S.B., Nasmyth K. (2000). Functional genomics identifies monopolin: a kinetochore protein required for segregation of homologs during meiosis I. Cell.

[bib34] Uhlmann F., Lottspeich F., Nasmyth K. (1999). Sister-chromatid separation at anaphase onset is promoted by cleavage of the cohesin subunit Scc1. Nature.

[bib35] Xu Z., Cetin B., Anger M., Cho U.S., Helmhart W., Nasmyth K., Xu W. (2009). Structure and function of the PP2A-shugoshin interaction. Mol. Cell.

[bib36] Yu H.G., Koshland D. (2005). Chromosome morphogenesis: condensin-dependent cohesin removal during meiosis. Cell.

