# Supplementary material for: Rec8 Phosphorylation by Casein Kinase 1 and Cdc7-Dbf4 Kinase Regulates Cohesin Cleavage by Separase during Meiosis
Source: Dev Cell. 2010 Mar 16;18(3):397–409. doi: 10.1016/j.devcel.2010.01.014 (PMC2994640; doi:10.1016/j.devcel.2010.01.014)
Supplement: Document S1. Seven Figures and Two Tables [file mmc1.pdf]

Developmental Cell *18*

Supplemental Information

Rec8 Phosphorylation by Casein Kinase 1

and Cdc7-Dbf4 Kinase Regulates

Cohesin Cleavage by Separase during Meiosis

Vittorio L. Katis, Jesse J. Lipp, Richard Imre, Aliona Bogdanova, Elwy Okaz, Bianca Habermann, Karl Mechtler, Kim Nasmyth, and Wolfgang Zachariae

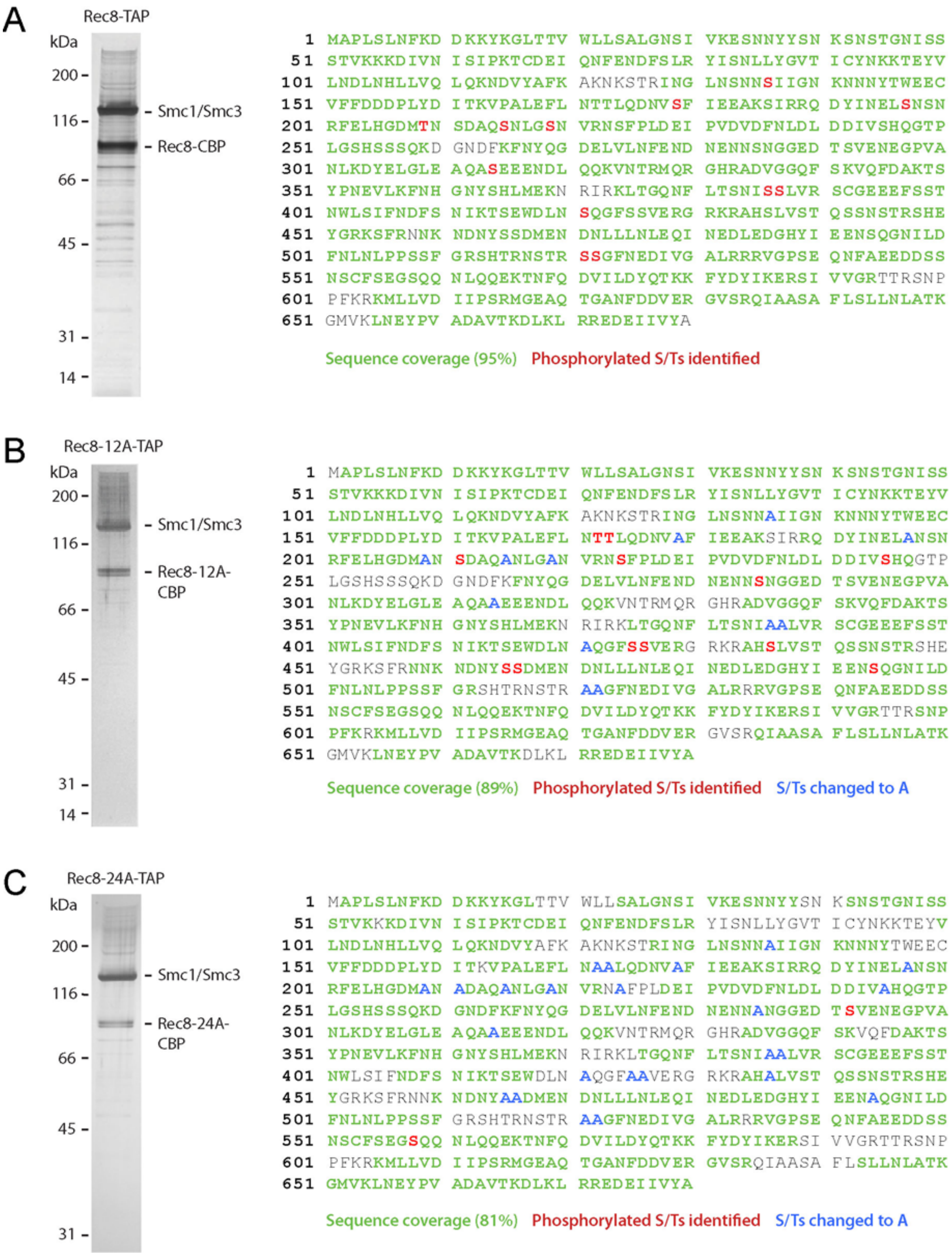

Figure S1. **Identification of Rec8 Residues Phosphorylated in Vivo (related to Figure 1)**
(A-C) Rec8 was isolated by tandem affinity purification (TAP) from *REC8-TAP* (K11905), *rec8-12A-TAP* (K14815), and *rec8-24A-TAP* (K15014) cells arrested in metaphase I due to meiotic depletion of Cdc20 (P_{CLB2}-*CDC20)*. Purified proteins were analyzed by SDS-PAGE and mass spectrometry. Left: silver-stained protein gels. CBP designates the calmodulin-binding peptide remaining on Rec8 after the TAP procedure. Right: Rec8 sequences with peptide sequence coverage (green), phosphorylated serine or threonine (red), and substitutions by alanine (blue). Analysis of Rec8-TAP is shown in (A), Rec8-12A-TAP in (B), and Rec8-24A-TAP in (C).

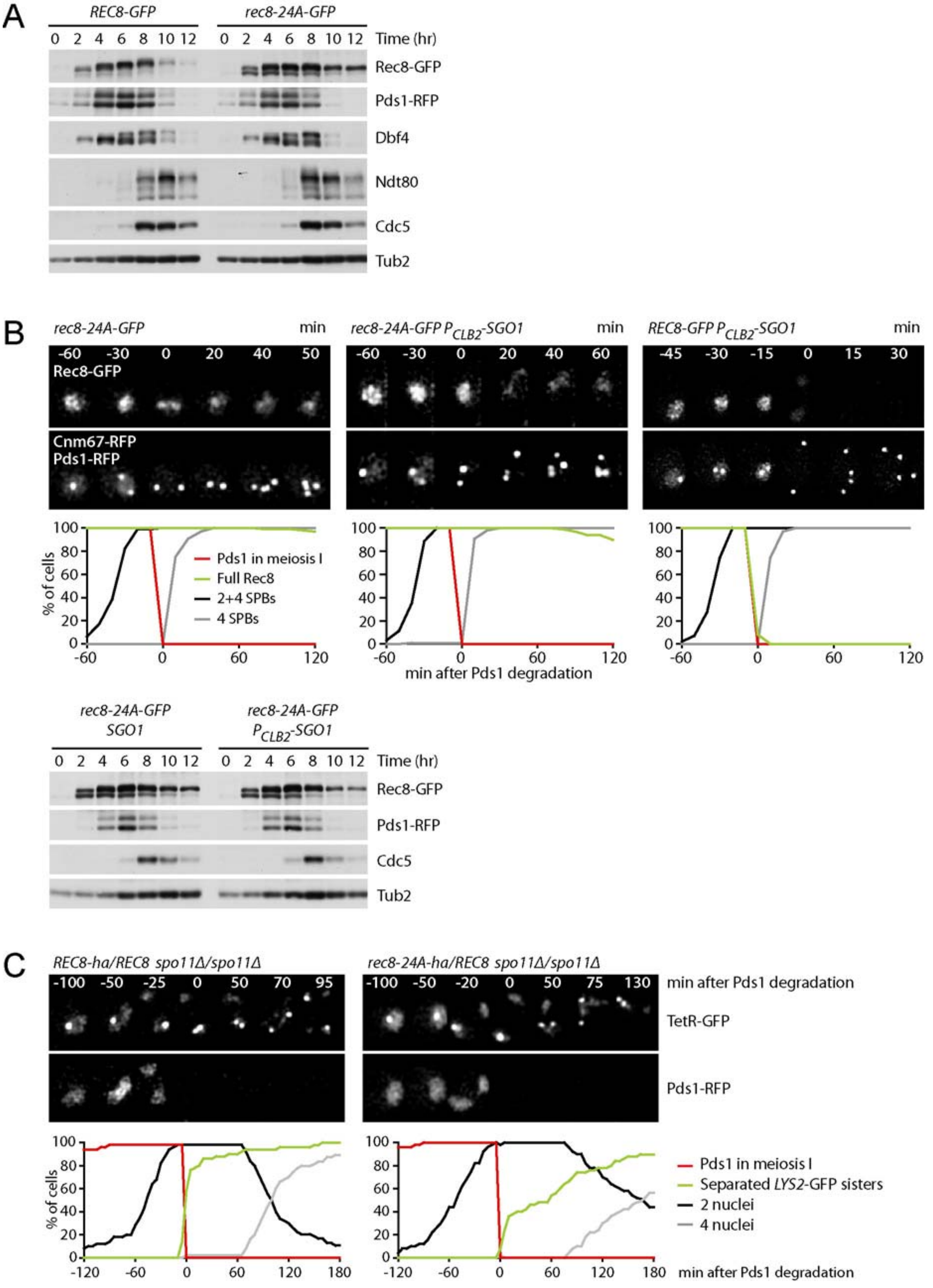

Figure S2. Analysis of *rec8-24A* Mutants (related to Figures 1 and 2)
(A) Progression through meiosis. *REC8-GFP* (Z12781) and *rec8-24A-GFP* (Z12782) strains containing Pds1-RFP and Cnm67-RFP at SPBs were induced to enter meiosis at 30°C. Aliquots were withdrawn for live-imaging (see Figure 1C) and samples collected every two hours were used to prepare protein extracts. Immunoblot analysis of protein levels is shown.
(B) Depletion of Sgo1 has no major effect on the persistence of Rec8-24A beyond meiosis I. *rec8-24A-GFP* (Z12782), *rec8-24A-GFP* P_{CLB2}-*SGO1* (Z16151), and *REC8-GFP* P_{CLB2}-*SGO1* (Z16150) cells containing Cnm67-RFP at SPBs and Pds1-RFP (fluorescence detectable only in meiosis I) were induced to enter meiosis at 30°C. P_{CLB2}-*SGO1* is expressed only in mitosis. Aliquots were subjected to live-imaging and samples collected every two hours were used to prepare protein extracts. Top: timelapse series with time (min) after Pds1 degradation in meiosis I. Middle: the presence of Pds1-RFP (meiosis I, red), nuclear Rec8-GFP (green), two or four SPBs (black), and four SPBs (grey) was quantified every 10 min in 100 cells, in which Pds1 degradation was set to t=0. Bottom: immunoblot analysis of protein levels.
(C) Elimination of recombination restores the meiosis I division in *rec8-24A/REC8* heterozygotes. TetR-GFP and Pds1-RFP were imaged in meiosis at 30°C in *spo11Δ* strains heterozygous for *LYS2-tetO* and *REC8-ha/REC8* (Z15787) or *rec8-24A-ha/REC8* (Z15788). TetR-GFP labels the nucleus (diffuse signal) and the sister chromatids of one chromosome II homolog (dots). Top: timelapse series. Bottom: the presence of Pds1-RFP (meiosis I, red), two *LYS2*-GFP dots (loss of arm cohesion, green), two nuclei (black), and four nuclei (grey) was quantified every 5 min in 100 cells, in which Pds1 degradation was set to t=0.

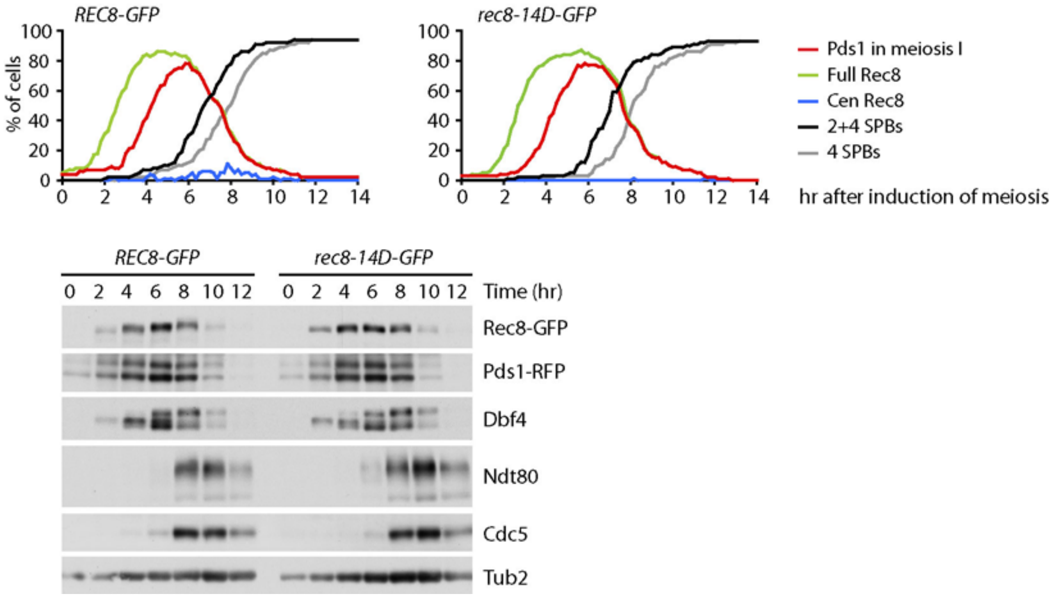

Figure S3. Progression of *rec8-14D* Mutants Through Meiosis (related to Figure 3)
REC8-GFP (Z12781) and *rec8-14D-GFP* (Z12783) cells containing Pds1-RFP and Cnm67-RFP at SPBs were induced to enter meiosis at 30°C. Aliquots were subjected to live-imaging (see also Figure 3C) and samples collected every two hours were used to prepare protein extracts. Top: the presence of Pds1-RFP (meiosis I, red), nuclear Rec8-GFP (entire chromatin, green; centromeric, green), two or four SPBs (black), and four SPBs (grey) was quantified every 10 min after induction of meiosis (t=0) in 100 cells. Bottom: immunoblot analysis of protein levels.

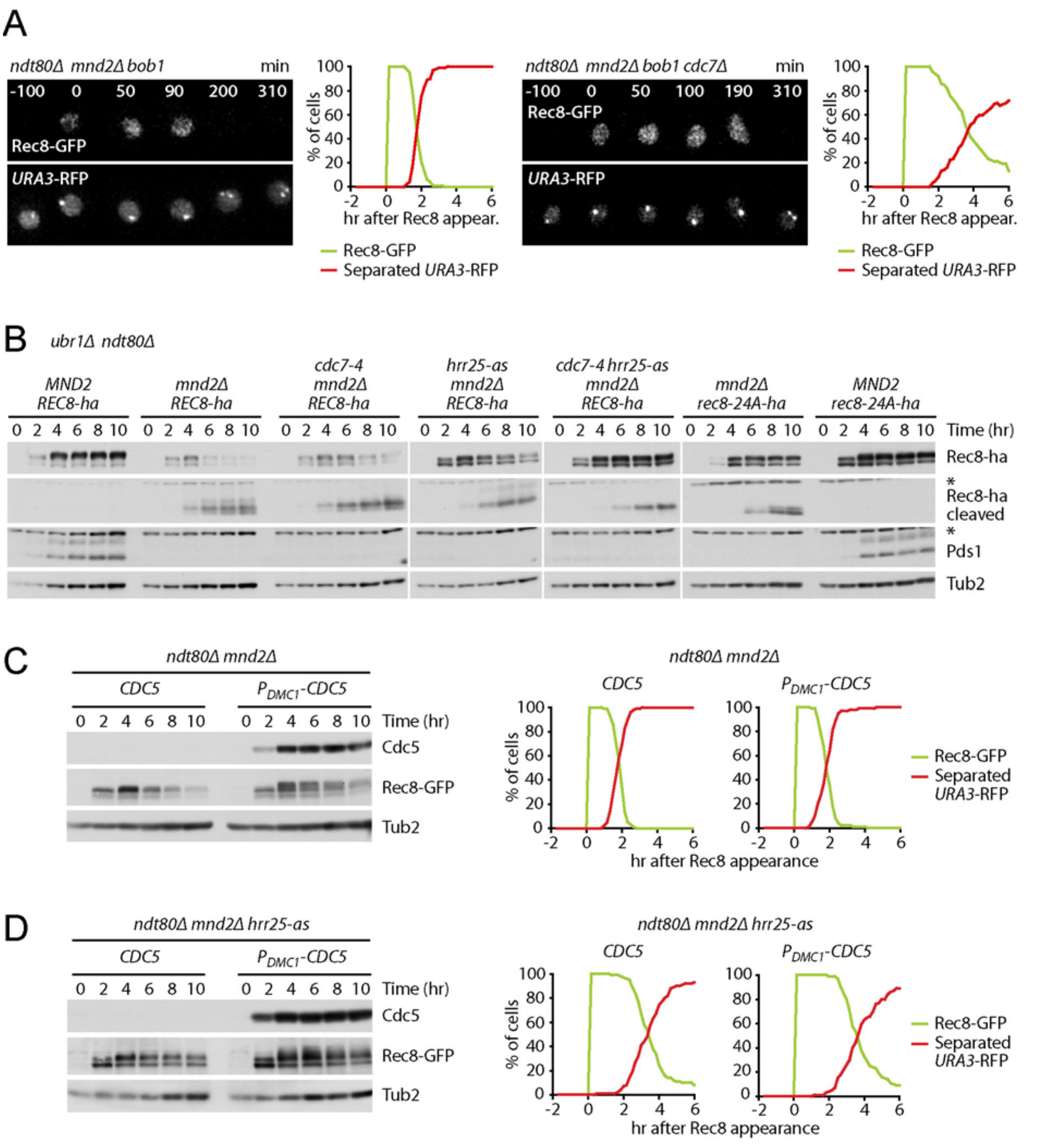

Figure S4. Analysis of Rec8 Destruction During Prophase I in *ndt80Δ mnd2Δ* Cells (related to Figure 6)

(A) Deletion of *CDC7* delays Rec8 destruction. *REC8-GFP ndt80Δ mnd2Δ bob1* cells containing TetR-RFP, heterozygous *URA3-tetO*, and either *CDC7* (Z15948) or *cdc7Δ* (Z15949) were imaged in meiosis at 30°C. The *bob1* mutation bypasses Cdc7's essential role in DNA replication (Hardy et al., 1997). Panels: timelapse series. Graphs: the presence of nuclear Rec8-GFP (green) and separated *URA3*-RFP sister sequences (red) was quantified every 10 min in 100 cells, in which Rec8 appearance was set to t=0.

(B) Immunoblot detection of Rec8-ha, cleavage products, Pds1, and tubulin in protein extracts from *ubr1Δ ndt80Δ* strains entering meiosis (31°C, 1NM-PP1). *REC8-ha* cells contain *MND2* (Z16025), *mnd2Δ* (Z4388), *mnd2Δ cdc7-4* (Z16027), *mnd2Δ hrr25-as* (Z16026), or *mnd2Δ cdc7-4 hrr25-as* (Z16135). *rec8-24A-ha* cells contain *mnd2Δ* (Z16220) or *MND2* (Z16221). Asterisks mark nonspecific bands.

(C and D) Strains with or without a construct expressing *CDC5* from the early meiosis-specific *DMC1* promoter (P_{DMC1}-*CDC5*) were induced to enter meiosis at 30°C. All strains contain Rec8-GFP, TetR-RFP, and heterozygous *URA3-tetO*. Aliquots were subjected to live-imaging and samples taken every two hours were used to prepare protein extracts. Panels: immunoblot analysis of protein levels. Graphs: analysis of Rec8-GFP and separated *URA3* sister sequences as in (A). (C) *ndt80Δ mnd2Δ* cells without (Z14432) or with (Z15213) P_{DMC1}-*CDC5*. (D) *ndt80Δ mnd2Δ hrr25-as* cells without (Z14981) or with (Z15163) P_{DMC1}-*CDC5* in the presence of 1NM-PP1.

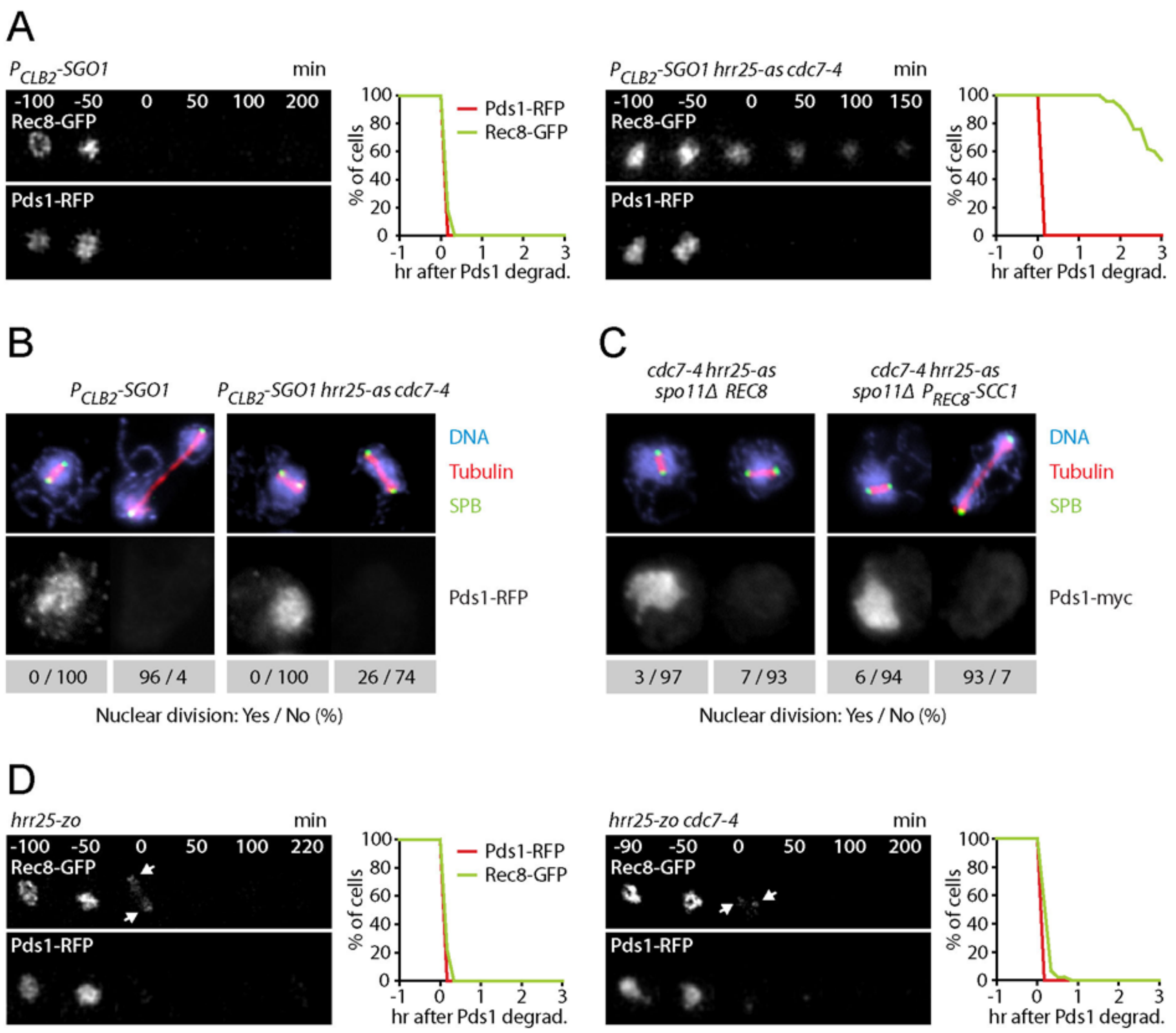

Figure S5. Analysis of Cohesin Cleavage at the Onset of Anaphase I (related to Figure 7)
(A and B) Inactivation of Hrr25 and DDK prevents Rec8 cleavage in the absence of Sgo1. P_{CLB2}-*SGO1* (Z16178) and P_{CLB2}-*SGO1 hrr25-as cdc7-4* (Z16157) cells with Rec8-GFP and Pds1-RFP were induced to enter meiosis (31°C, 1NM-PP1). P_{CLB2}-*SGO1* is expressed only in mitosis. Cells were analyzed by live-imaging and immunofluorescence microscopy of fixed cells. (A) Live-imaging. Panels: timelapse series with time (min) after Pds1 degradation in meiosis I. Graphs: the presence of nuclear Rec8-GFP (green) and Pds1-RFP (meiosis I, red) was quantified every 10 min in 50 cells, in which Pds1 degradation was set to t=0. (B) Fixed cells were stained for DNA, tubulin, Tub4/γ-tubulin at SPBs, and Pds1-RFP. Nuclear division was quantified in Pds1-positive and Pds1-negative cells with two SPBs (meiosis I, n=50). Due to bi-orientation of sister centromeres, the meiosis I division is blocked in *cdc7-4* and *hrr25-as* single mutants but can be restored by depleting Sgo1 (Matos et al., 2008; Petronczki et al., 2006). The absence of a meiosis I division in most *cdc7-4 hrr25-as* double mutant cells lacking Sgo1 indicates a strong delay in Rec8 cleavage.
(C) Cleavage of Scc1 at meiosis I does not require Hrr25 or DDK activity. *cdc7-4 hrr25-as spo11Δ PDS1-myc18* cells expressing either *REC8* (Z15888) or *SCC1* (P_{REC8}-*SCC1*, Z15887) from the *REC8* promoter were induced to enter meiosis (31°C, 1NM-PP1). Fixed cells were stained for DNA, tubulin, Tub4/γ-tubulin at SPBs, and Pds1-myc. Nuclear division was quantified in Pds1-positive and Pds1-negative cells with two SPBs (meiosis I, n=100).
(D) Rec8 cleavage requires the kinase activity but not binding to monopolin of Hrr25. Rec8-GFP and Pds1-RFP were imaged during meiosis at 31°C in *hrr25-zo* (Z15446) and *hrr25-zo cdc7-4* (Z15443) cells. The *hrr25-zo* mutation prevents Hrr25's binding to the monopolin subunit Mam1 (Petronczki et al., 2006). Panels: timelapse series with time (min) after Pds1 degradation in meiosis I. Arrows mark centromeric Rec8 in anaphase I. Graphs: the presence of nuclear Rec8-GFP (green) and Pds1-RFP (meiosis I, red) was analyzed as in (A).

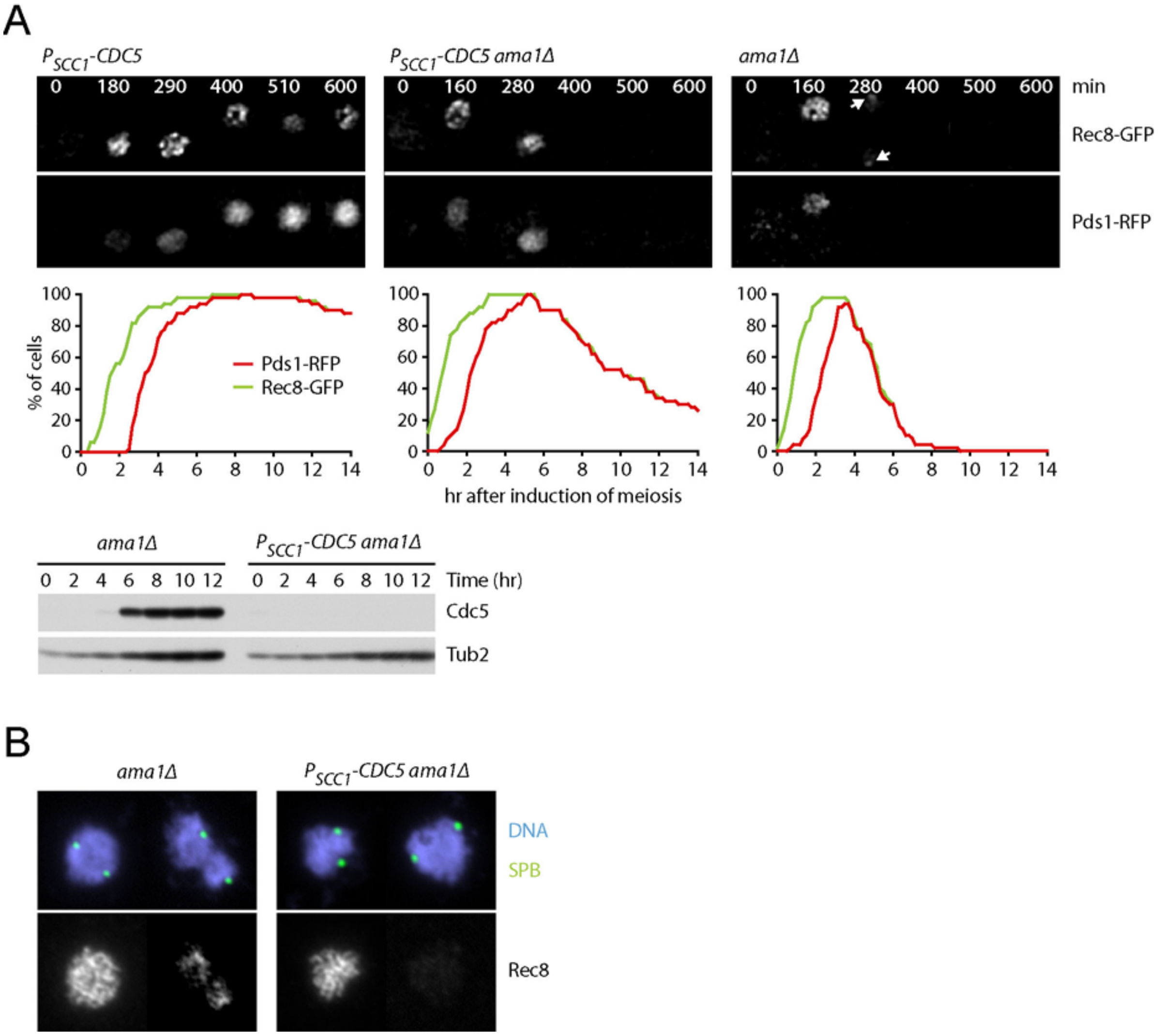

Figure S6. Deletion of *AMA1* Restores Pds1 Degradation and Rec8 Cleavage in Cells Lacking Cdc5 (related to Figure 7)

(A) P_{SCC1}-*CDC5* (Z16253), P_{SCC1}-*CDC5 ama1Δ* (Z15538), and *ama1Δ* (Z16346) cells containing Rec8-GFP and Pds1-RFP were induced to enter meiosis at 30°C. P_{SCC1}-*CDC5* is expressed only in mitosis. Aliquots were subjected to live-imaging and samples collected every two hours were used to prepare protein extracts. Top: timelapse series with time (min) after induction of meiosis. Arrows mark centromeric Rec8. Middle: the presence of nuclear Rec8-GFP (green) and Pds1-RFP (meiosis I, red) was quantified every 10 min after induction of meiosis (t=0) in 50 cells. Bottom: Depletion of Cdc5 was analyzed by immunoblotting.

(B) Cdc5 is required for the persistence of centromeric cohesin at anaphase I in *ama1Δ* cells. Chromosome spreads were prepared from *ama1Δ* (Z15849) and P_{SCC1}-*CDC5 ama1Δ* (Z15850) cells entering meiosis at 30°C. Panels show staining of DNA, Tub4/γ-tubulin at SPBs, and Rec8 on spreads containing two SPBs (metaphase I or anaphase I). In the case of *ama1Δ* cells, these spreads show either full or centromeric Rec8 staining. In the case of P_{SCC1}-*CDC5 ama1Δ* cells, they show either full or no Rec8 staining.

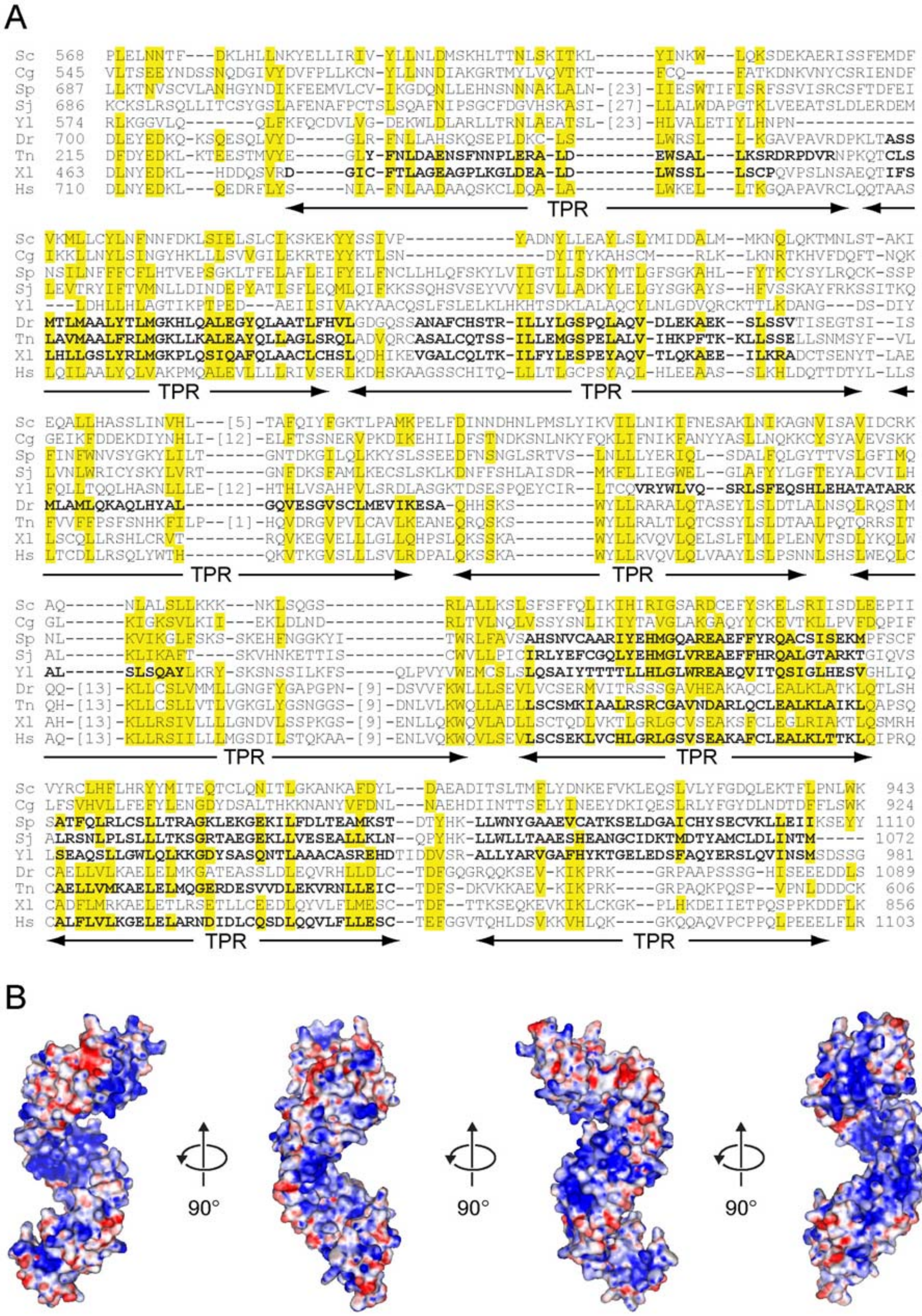

Figure S7. Structural Model of the Conserved C-Terminal Half of Separase's Noncatalytic Domain (related to Figure 8)

(A and B) To investigate whether the noncatalytic domain of *Saccharomyces cerevisiae* separase contains features suitable for binding negatively charged ligands such as phosphorylated Rec8, we sought to generate a structural model. To identify evolutionary conserved regions, we performed PSI-BLAST searches (Altschul et al., 1997) with default parameters against the nonredundant protein database. Residues 560-1000 retrieve the corresponding region from separases of various organisms indicating stronger sequence conservation in the C-terminal than in the N-terminal half of the noncatalytic domain. Since the C-terminal half contains multiple copies of the tetratricopeptide repeat (TPR) and retrieves different TPR proteins, it was modelled onto the structure of the TPR protein O-linked GlcNAc transferase (OGT). (A) Multiple sequence alignment of residues

568-943 of *S. cerevisiae* separase (Sc, NP_011612) with sequences from *Candida glabrata* (Cg, XP_446503), *Schizosaccharomyces pombe* (Sp, NP_587903), *Schizosaccharomyces japonicus* (Sj, XP_002173750), *Yarrowia lipolytica* (Yl, XP_501622), *Danio rerio* (Dr, XP_001337869), *Tetraodon nigroviridis* (Tn, CAG06749), *Xenopus laevis* (Xl, AAH70872), and *Homo sapiens* (Hs, NP_036423). Conserved residues are highlighted in yellow. TPR repeats predicted by TPRpred (Karpenahalli et al., 2007) are shown in bold and those identified by profile-profile comparisons (hhpred; Soeding, 2005) are indicated below the alignment. The multiple sequence alignment was generated manually based on pair-wise BLAST alignments. (B) Modelling of the conserved C-terminal half of the noncatalytic domain from *S. cerevisiae* separase on the structure of the TPR domain of OGT (pdb code 1W3B; Jinek et al., 2004). The model was generated with SwissModel (http://swissmodel.expasy.org) based on a pair-wise alignment of yeast separase with OGT. The electrostatic surface potential was calculated using APBS (Baker et al., 2001) with default parameters and displayed in PyMOL (http://www.pymol.org). Blue and red represent regions of positive and negative electrostatic potential (± 20 kT/*e*), respectively. We speculate that the extended, positively charged surface along the TPR superhelix binds phosphate groups in Rec8.

Table S1. Proteins Identified in the Rec8-TAP Purification (related to Figure 1)

Protein	Sequence coverage	kDa	Annotation
Rec8	95%	77	Cohesin subunit
Smc1	69%	142	Cohesin subunit
Smc3	68%	141	Cohesin subunit
Pds5	41%	147	Cohesin subunit
Hrr25	38%	58	Casein kinase 1δ/ε
Dmc1	35%	37	Meiotic recombinase
Cdc5	33%	81	Polo-like kinase
Scc3	27%	134	Cohesin subunit
Hop1	19%	70	Meiotic recombination

Proteins isolated by tandem affinity purification (TAP) from extracts of metaphase I-arrested P_{CLB2}-*CDC20 REC8-TAP* cells (K11905) were identified by mass spectrometry and ranked by peptide sequence coverage.

Table S2. ***Saccharomyces cerevisiae*** **Strains Used in this Study**

Strain	Genotype*
K8965	W303 MATα, esp1-1, ura3::tetOx240::URA3, leu2::P_{URA3}-tetR-GFP::LEU2, P_{GAL}-FLAG-ESP1-CBP::KlTRP1
K8967	W303 MATα, esp1-1, ura3::tetOx240::URA3, leu2::P_{URA3}-tetR-GFP::LEU2, P_{GAL}-FLAG-esp1-C1531A-CBP::KlTRP1
K11905	MATa/MATα, REC8-ha2-TAP::URA3/REC8-ha2-TAP::URA3, cdc20::P_{CLB2}-CDC20::kanMX6/cdc20::P_{CLB2}-CDC20::kanMX6, SPC42-GFP::HIS3MX6/SPC42
K14815	MATa/MATα, rec8-12A-ha2-TAP::URA3::LEU2::rec8Δ::kanMX4/rec8-12A-ha2-TAP::URA3::LEU2::rec8Δ::kanMX4, cdc20::P_{CLB2}-CDC20::kanMX6/cdc20::P_{CLB2}-CDC20::kanMX6, SPC42-GFP::HIS3MX6/SPC42
K14907	MATa/MATα, REC8-ha3::LEU2::rec8Δ::kanMX4/REC8-ha3::LEU2::rec8Δ::kanMX4, PDS1-myc18::KlTRP1/PDS1-myc18::KlTRP1
K15010	MATa/MATα, rec8-24A-ha3::LEU2::rec8Δ::kanMX4/rec8-24A-ha3::LEU2::rec8Δ::kanMX4, PDS1-myc18::KlTRP1/PDS1-myc18::KlTRP1
K15014	MATa/MATα, rec8-24A-ha2-TAP::URA3::LEU2::rec8Δ::kanMX4/rec8-24A-ha2-TAP::URA3::LEU2::rec8Δ::kanMX4, cdc20::P_{CLB2}-CDC20::kanMX6/cdc20::P_{CLB2}-CDC20::kanMX6, SPC42-GFP::HIS3MX6/SPC42
K15176	MATa/MATα, REC8-ha3::LEU2::rec8Δ::kanMX4/REC8, PDS1-myc18::KlTRP1/PDS1-myc18::KlTRP1, lys2::tetOx240::URA3/lys2::tetOx240::URA3, leu2::P_{URA3}-tetR-GFP::LEU2/leu2::P_{URA3}-tetR-GFP::LEU2
K15178	MATa/MATα, rec8-24A-ha3::LEU2::rec8Δ::kanMX4/REC8, PDS1-myc18::KlTRP1/PDS1-myc18::KlTRP1, lys2::tetOx240::URA3/lys2::tetOx240::URA3, leu2::P_{URA3}-tetR-GFP::LEU2/leu2::P_{URA3}-tetR-GFP::LEU2
K15246	MATa/MATα, REC8-ha3::HIS3::rec8Δ::kanMX4/rec8Δ::kanMX4, PDS1-myc18::KlTRP1/PDS1-myc18::KlTRP1, ura3::tetOx240::URA3/ura3, leu2::P_{URA3}-tetR-GFP::LEU2/leu2::P_{URA3}-tetR-GFP::LEU2
K15247	MATa/MATα, rec8-14D-ha3::HIS3:rec8Δ::kanMX4/rec8Δ::kanMX4, PDS1-myc18::KlTRP1/PDS1-myc18::KlTRP1, ura3::tetOx240::URA3/ura3, leu2::P_{URA3}-tetR-GFP::LEU2/leu2::P_{URA3}-tetR-GFP::LEU2
K15444	MATa/MATα, REC8-ha3::HIS3:rec8Δ::kanMX4/rec8Δ::kanMX4, PDS1-myc18::KlTRP1/PDS1-myc18::KlTRP1, ura3::tetOx240::URA3/ura3, leu2::P_{URA3}-tetR-GFP::LEU2/leu2::P_{URA3}-tetR-GFP::LEU2
K15445	MATa/MATα, rec8-14D-ha3::HIS3:rec8Δ::kanMX4/rec8Δ::kanMX4, PDS1-myc18::KlTRP1/PDS1-myc18::KlTRP1, ura3::tetOx240::URA3/ura3, leu2::P_{URA3}-tetR-GFP::LEU2/leu2::P_{URA3}-tetR-GFP::LEU2
K15446	MATa/MATα, spo11Δ::KlTRP1/spo11Δ::KlTRP1, REC8-ha3::HIS3::rec8Δ::kanMX4/rec8Δ::kanMX4, PDS1-myc18::KlTRP1/PDS1-myc18::KlTRP1, ura3::tetOx240::URA3/ura3, leu2::P_{URA3}-tetR-GFP::LEU2/leu2::P_{URA3}-tetR-GFP::LEU2
K15447	MATa/MATα, spo11Δ::KlTRP1/spo11Δ::KlTRP1, rec8-14D-ha3::HIS3::rec8Δ::kanMX4/rec8Δ::kanMX4, PDS1-myc18::KlTRP1/PDS1-myc18::KlTRP1, ura3::tetOx240::URA3/ura3, leu2::P_{URA3}-tetR-GFP::LEU2/leu2::P_{URA3}-tetR-GFP::LEU2
K15448	MATa/MATα, mam1Δ::natMX4/mam1Δ::natMX4, REC8-ha3::HIS3::rec8Δ::kanMX4/rec8Δ::kanMX4, PDS1-myc18::KlTRP1/PDS1-myc18::KlTRP1, ura3::tetOx240::URA3/ura3, leu2::P_{URA3}-tetR-GFP::LEU2/leu2::P_{URA3}-tetR-GFP::LEU2
K15449	MATa/MATα, mam1Δ::natMX4/mam1Δ::natMX4, rec8-14D-ha3::HIS3::rec8Δ::kanMX4/rec8Δ::kanMX4, PDS1-myc18::KlTRP1/PDS1-myc18::KlTRP1, ura3::tetOx240::URA3/ura3, leu2::P_{URA3}-tetR-GFP::LEU2/leu2::P_{URA3}-tetR-GFP::LEU2

K15450	MATa/MATα, mam1Δ::natMX4/mam1Δ::natMX4, spo11Δ::KlTRP1/spo11Δ::KlTRP1, REC8-ha3::HIS3::rec8Δ::kanMX4/rec8Δ::kanMX4, PDS1-myc18::KlTRP1/PDS1-myc18::KlTRP1, ura3::tetOx240::URA3/ura3, leu2::P_{URA3}-tetR-GFP::LEU2/leu2::P_{URA3}-tetR-GFP::LEU2
K15451	MATa/MATα, mam1Δ::natMX4/mam1Δ::natMX4, spo11Δ::KlTRP1/spo11Δ::KlTRP1, rec8-14D-ha3::HIS3::rec8Δ::kanMX4/rec8Δ::kanMX4, PDS1-myc18::KlTRP1/PDS1-myc18::KlTRP1, ura3::tetOx240::URA3/ura3, leu2::P_{URA3}-tetR-GFP::LEU2/leu2::P_{URA3}-tetR-GFP::LEU2
K15584	MATa/MATα, REC8-ha3::LEU2::rec8Δ::kanMX4/REC8-myc9::URA3, PDS1-myc18::KlTRP1/PDS1-myc18::KlTRP1, leu2::P_{GAL1}-NDT80::LEU2/leu2::P_{GAL1}-NDT80::LEU2, ndt80Δ::natMX4/ndt80Δ::natMX4, his3::P_{GDP1}-GAL4-ER::HIS3/his3
K15585	MATa/MATα, rec8-24A-ha3::LEU2::rec8Δ::kanMX4/REC8-myc9::URA3, PDS1-myc18::KlTRP1/PDS1-myc18::KlTRP1, leu2::P_{GAL1}-NDT80::LEU2/leu2::P_{GAL1}-NDT80::LEU2, ndt80Δ::natMX4/ndt80Δ::natMX4, his3::P_{GDP1}-GAL4-ER::HIS3/his3
K15991	MATa/MATα, REC8-ha3::LEU2::rec8Δ::kanMX4/rec8Δ::kanMX4, SGO1-myc9::KlTRP1/SGO1-myc9::KlTRP1, NDC10-ha6::HIS3MX6/NDC10-ha6::HIS3MX6
K15992	MATa/MATα, rec8-14D-ha3::LEU2::rec8Δ::kanMX4/rec8Δ::kanMX4, SGO1-myc9::KlTRP1/SGO1-myc9::KlTRP1, NDC10-ha6::HIS3MX6/NDC10-ha6::HIS3MX6
K15993	MATa/MATα, REC8-ha3::LEU2::rec8Δ::kanMX4/rec8Δ::kanMX4, RTS1-myc9::KlTRP1/RTS1-myc9::KlTRP1, NDC10-ha6::HIS3MX6/NDC10-ha6::HIS3MX6
K15994	MATa/MATα, rec8-14D-ha3::LEU2::rec8Δ::kanMX4/rec8Δ::kanMX4, RTS1-myc9::KlTRP1/RTS1-myc9::KlTRP1, NDC10-ha6::HIS3MX6/NDC10-ha6::HIS3MX6
K16271	MATa/MATα, rec8-7D-I-ha3::HIS3::rec8Δ::kanMX4/rec8Δ::kanMX4, PDS1-myc18::KlTRP1/PDS1-myc18::KlTRP1, ura3::tetOx240::URA3/ura3, leu2::P_{URA3}-tetR-GFP::LEU2/leu2::P_{URA3}-tetR-GFP::LEU2
K16272	MATa/MATα, rec8-7D-II-ha3::HIS3::rec8Δ::kanMX4/rec8Δ::kanMX4, PDS1-myc18::KlTRP1/PDS1-myc18::KlTRP1, ura3::tetOx240::URA3/ura3, leu2::P_{URA3}-tetR-GFP::LEU2/leu2::P_{URA3}-tetR-GFP::LEU2
K16420	MATa/MATα, rec8-4D-ha3::HIS3::rec8Δ::kanMX4/rec8Δ::kanMX4, PDS1-myc18::KlTRP1/PDS1-myc18::KlTRP1, ura3::tetOx240::URA3/ura3, leu2::P_{URA3}-tetR-GFP::LEU2/leu2::P_{URA3}-tetR-GFP::LEU2
K16421	MATa/MATα, rec8-3D-ha3::HIS3::rec8Δ::kanMX4/rec8Δ::kanMX4, PDS1-myc18::KlTRP1/PDS1-myc18::KlTRP1, ura3::tetOx240::URA3/ura3, leu2::P_{URA3}-tetR-GFP::LEU2/leu2::P_{URA3}-tetR-GFP::LEU2
K17066	MATa/MATα, REC8-ha3::LEU2::rec8Δ::kanMX4/REC8-ha3::LEU2::rec8Δ::kanMX4, ndt80Δ::HIS3/ndt80Δ::HIS3
K17068	MATa/MATα, REC8-ha3::LEU2::rec8Δ::kanMX4/REC8-ha3::LEU2::rec8Δ::kanMX4, ndt80Δ::natMX4/ndt80Δ::natMX4, cdc7-4/cdc7-4, hrr25-as1::HIS3::hrr25Δ::kanMX4/hrr25-as1::HIS3::hrr25Δ::kanMX4
K17069	MATa/MATα, rec8-14D-ha3::LEU2::rec8Δ::kanMX4/rec8-14D-ha3::LEU2::rec8Δ::kanMX4, ndt80Δ::natMX4/ndt80Δ::natMX4, cdc7-4/cdc7-4, hrr25-as1::HIS3::hrr25Δ::kanMX4/hrr25-as1::HIS3::hrr25Δ::kanMX4
Z4388	MATa/MATα, REC8-ha3::LEU2::rec8Δ::kanMX4/REC8, ubr1Δ::KlTRP1/ubr1Δ::KlTRP1, ndt80::HIS3/ndt80::HIS3, mnd2Δ::CaURA3/mnd2Δ::CaURA3
Z5620	MATa/MATα, cdc20::P_{CLB2}-CDC20::kanMX6/cdc20::P_{CLB2}-CDC20::kanMX6
Z7271	MATa/MATα, spo11::hisG-URA3-hisG/spo11::hisG-URA3-hisG, PDS1-myc18::KlTRP1/PDS1, CENV::tetOx224::HIS3/CENV, leu2::P_{URA3}-tetR-GFP::LEU2/leu2
Z7532	MATa/MATα, cdc20::P_{CLB2}-CDC20::kanMX6/cdc20::P_{CLB2}-CDC20::kanMX6, REC8-ha3::URA3/REC8-ha3::URA3
Z8225	MATa/MATα, spo11Δ::HIS3MX6/spo11Δ::HIS3MX6, REC8-ha3::URA3/REC8-ha3::URA3, PDS1-myc18::KlTRP1/PDS1-myc18::KlTRP1, ura3::tetOx240::URA3/ura3, leu2::P_{URA3}-tetR-GFP::LEU2/leu2

Z8444	*MATa/MATα, spo11Δ::KlTRP1/spo11Δ::KlTRP1, rec8Δ::kanMX4::P_{REC8}-SCC1-ha3::LEU2/rec8Δ::kanMX4::P_{REC8}-SCC1-ha3::LEU2, PDS1-myc18::KlTRP1/PDS1-myc18::KlTRP1, ura3::tetOx240::URA3/ura3, leu2::P_{URA3}-tetR-GFP::LEU2/leu2*
Z8535	*MATa/MATα, cdc20::P_{CLB2}-CDC20::kanMX6/cdc20::P_{CLB2}-CDC20::kanMX6, CDC7-myc9::KlTRP1/CDC7-myc9::KlTRP1*
Z8536	*MATa/MATα, cdc20::P_{CLB2}-CDC20::kanMX6/cdc20::P_{CLB2}-CDC20::kanMX6, REC8-ha3::URA3/REC8-ha3::URA3, CDC7-myc9::KlTRP1/CDC7-myc9::KlTRP1*
Z9052	*MATa/MATα, bob1::natMX4/bob1::natMX4, REC8-ha3::URA3/REC8-ha3::URA3, PDS1-myc18/PDS1, ura3::tetOx224::URA3/ura3, leu2::P_{URA3}-tetR-GFP::LEU2/leu2*
Z9053	*MATa/MATα, bob1::natMX4/bob1::natMX4, PDS1-myc18/PDS1, ura3::tetOx224::URA3/ura3, leu2::P_{URA3}-tetR-GFP::LEU2/leu2*
Z9320	*MATa/MATα, cdc20::P_{CLB2}-CDC20::kanMX6/cdc20::P_{CLB2}-CDC20::kanMX6, cdc5::P_{SCC1}-CDC5::kanMX4/cdc5::P_{SCC1}-CDC5::kanMX4, REC8-ha3::URA3/REC8-ha3::URA3, CDC7-myc9::KlTRP1/CDC7-myc9::KlTRP1*
Z9341	*MATa/MATα, bob1::natMX4/bob1::natMX4, cdc7Δ::kanMX4/cdc7Δ::kanMX4, REC8-ha3::URA3/REC8-ha3::URA3, PDS1-myc18/PDS1, ura3::tetOx224::URA3/ura3, leu2::P_{URA3}-tetR-GFP::LEU2/leu2*
Z10266	*MATa/MATα, cdc20::P_{CLB2}-CDC20::kanMX6/cdc20::P_{CLB2}-CDC20::kanMX6, HRR25::HIS3::hrr25Δ::kanMX4/HRR25::HIS3::hrr25Δ::kanMX4, REC8-ha3::URA3/REC8-ha3::URA3, CDC7-myc9::KlTRP1/CDC7-myc9::KlTRP1*
Z10271	*MATa/MATα, cdc20::P_{CLB2}-CDC20::kanMX6/cdc20::P_{CLB2}-CDC20::kanMX6, HRR25::HIS3::hrr25Δ::kanMX4/HRR25::HIS3::hrr25Δ::kanMX4, CDC7-myc9::KlTRP1/CDC7-myc9::KlTRP1*
Z10274	*MATa/MATα, cdc20::P_{CLB2}-CDC20::kanMX6/cdc20::P_{CLB2}-CDC20::kanMX6, hrr25-as1::HIS3::hrr25Δ::kanMX4/hrr25-as1::HIS3::hrr25Δ::kanMX4, REC8-ha3::URA3/REC8-ha3::URA3, CDC7-myc9::KlTRP1/CDC7-myc9::KlTRP1*
Z12035	*MATa/MATα, REC8-ha3::LEU2::rec8Δ::kanMX4/REC8, PDS1-tdTomato::KlTRP1/PDS1-tdTomato::KlTRP1, lys2::tetOx240::URA3/lys2::tetOx240::URA3, leu2::P_{URA3}-tetR-GFP::LEU2/leu2::P_{URA3}-tetR-GFP::LEU2*
Z12036	*MATa/MATα, rec8-24A-ha3::LEU2::rec8Δ::kanMX4/REC8, PDS1-tdTomato::KlTRP1/PDS1-tdTomato::KlTRP1, lys2::tetOx240::URA3/lys2::tetOx240::URA3, leu2::P_{URA3}-tetR-GFP::LEU2/leu2::P_{URA3}-tetR-GFP::LEU2*
Z12781	*MATa/MATα, REC8-GFP::LEU2::rec8Δ::kanMX4/REC8-GFP::LEU2::rec8Δ::kanMX4, PDS1-tdTomato::KlTRP1/PDS1-tdTomato::KlTRP1, CNM67-tdTomato::natMX4/CNM67-tdTomato::natMX4*
Z12782	*MATa/MATα, rec8-24A-GFP::LEU2::rec8Δ::kanMX4/rec8-24A-GFP::LEU2::rec8Δ::kanMX4, PDS1-tdTomato::KlTRP1/PDS1-tdTomato::KlTRP1, CNM67-tdTomato::natMX4/CNM67-tdTomato::natMX4*
Z12783	*MATa/MATα, rec8-14D-GFP::LEU2::rec8Δ::kanMX4/rec8-14D-GFP::LEU2::rec8Δ::kanMX4, PDS1-tdTomato::KlTRP1/PDS1-tdTomato::KlTRP1, CNM67-tdTomato::natMX4/CNM67-tdTomato::natMX4*
Z13861	*MATa/MATα, rec8-24A-ha3::LEU2::rec8Δ::kanMX4/rec8-24A-ha3::LEU2::rec8Δ::kanMX4, PDS1-tdTomato::KlTRP1/PDS1-tdTomato::KlTRP1, lys2::tetOx240::URA3/lys2::tetOx240::URA3, leu2::P_{URA3}-tetR-GFP::LEU2/leu2::P_{URA3}-tetR-GFP::LEU2*
Z14429	*MATa/MATα, ndt80::HIS3/ndt80::HIS3, REC8-GFP::URA3/REC8-GFP::URA3, ura3::tetOx224::URA3/ura3, leu2::P_{URA3}-tetR-tdTomato::LEU2/leu2*
Z14432	*MATa/MATα, ndt80::HIS3/ndt80::HIS3, mnd2Δ::kanMX4/mnd2Δ::kanMX4, REC8-GFP::URA3/REC8-GFP::URA3, ura3::tetOx224::URA3/ura3, leu2::P_{URA3}-tetR-tdTomato::LEU2/leu2*

Z14489	*MATa/MATα, ndt80::HIS3/ndt80::HIS3, rec8-24A-GFP::LEU2::rec8Δ::kanMX4/rec8-24A-GFP::LEU2::rec8Δ::kanMX4, ura3::tetOx224::URA3/ura3, leu2::*P_{URA3}*-tetR-tdTomato::LEU2/leu2*
Z14492	*MATa/MATα, ndt80::HIS3/ndt80::HIS3, mnd2Δ::kanMX4/mnd2Δ::kanMX4, rec8-24A-GFP::LEU2::kanMX4/rec8-24A-GFP::LEU2::kanMX4, ura3::tetOx224::URA3/ura3, leu2::*P_{URA3}*-tetR-tdTomato::LEU2/leu2*
Z14855	*MATa/MATα, REC8-ha3::LEU2::rec8Δ::kanMX4/REC8-ha3::LEU2::rec8Δ::kanMX4, SGO1-GFP::KlTRP1/SGO1-GFP::KlTRP1, MTW1-mCherry::hphMX4/MTW1-mCherry::hphMX4*
Z14860	*MATa/MATα, rec8-24A-ha3::LEU2::rec8Δ::kanMX4/rec8-24A-ha3::LEU2::rec8Δ::kanMX4, SGO1-GFP::KlTRP1/SGO1-GFP::KlTRP1, MTW1-mCherry::hphMX4/MTW1-mCherry::hphMX4*
Z14981	*MATa/MATα, ndt80Δ::natMX4/ndt80Δ::natMX4, mnd2Δ::hphMX4/mnd2Δ::hphMX4, hrr25-as1::HIS3::hrr25Δ::kanMX4/hrr25-as1::HIS3::hrr25Δ::kanMX4, REC8-GFP::URA3/REC8-GFP::URA3, ura3::tetOx224::URA3/ura3, leu2::*P_{URA3}*-tetR-tdTomato::LEU2/leu2*
Z15055	*MATa/MATα, HRR25::HIS3::hrr25Δ::kanMX4/HRR25::HIS3::hrr25Δ::kanMX4, cdc7-4/cdc7-4, REC8-GFP::URA3/REC8-GFP::URA3, PDS1-tdTomato::KlTRP1/PDS1-tdTomato::KlTRP1*
Z15058	*MATa/MATα, hrr25-as1::HIS3::hrr25Δ::kanMX4/hrr25-as1::HIS3::hrr25Δ::kanMX4, cdc7-4/cdc7-4, REC8-GFP::URA3/REC8-GFP::URA3, PDS1-tdTomato::KlTRP1/PDS1-tdTomato::KlTRP1*
Z15135	*MATa/MATα, HRR25::HIS3::hrr25Δ::kanMX4/HRR25::HIS3::hrr25Δ::kanMX4, REC8-GFP::URA3/REC8-GFP::URA3, PDS1-tdTomato::KlTRP1/PDS1-tdTomato::KlTRP1*
Z15138	*MATa/MATα, hrr25-as1::HIS3::hrr25Δ::kanMX4/hrr25-as1::HIS3::hrr25Δ::kanMX4, REC8-GFP::URA3/REC8-GFP::URA3, PDS1-tdTomato::KlTRP1/PDS1-tdTomato::KlTRP1*
Z15163	*MATa/MATα, ndt80Δ::natMX4/ndt80Δ::natMX4, mnd2Δ::hphMX4/mnd2Δ::hphMX4, trp1::*P_{DMC1}*-CDC5::TRP1/trp1::*P_{DMC1}*-CDC5::TRP1, hrr25-as1::HIS3::hrr25Δ::kanMX4/hrr25-as1::HIS3::hrr25Δ::kanMX4, REC8-GFP::URA3/REC8-GFP::URA3, ura3::tetOx224::URA3/ura3, leu2::*P_{URA3}*-tetR-tdTomato::LEU2/leu2*
Z15180	*MATa/MATα, ndt80::HIS3/ndt80::HIS3, mnd2Δ::kanMX4/mnd2Δ::kanMX4, REC8-GFP::TRP1::rec8Δ::kanMX4/REC8-GFP::TRP1::rec8Δ::kanMX4, ura3::tetOx224::URA3/ura3, leu2::*P_{URA3}*-tetR-tdTomato::LEU2/leu2*
Z15181	*MATa/MATα, ndt80::HIS3/ndt80::HIS3, mnd2Δ::kanMX4/mnd2Δ::kanMX4, rec8-14D-GFP::TRP1::rec8Δ::kanMX4/rec8-14D-GFP::TRP1::rec8Δ::kanMX4, ura3::tetOx224::URA3/ura3, leu2::*P_{URA3}*-tetR-tdTomato::LEU2/leu2*
Z15182	*MATa/MATα, ndt80::HIS3/ndt80::HIS3, mnd2Δ::kanMX4/mnd2Δ::kanMX4, hrr25-as1::HIS3::hrr25Δ::kanMX4/hrr25-as1::HIS3::hrr25Δ::kanMX4, cdc7-4/cdc7-4, rec8-14D-GFP::TRP1::rec8Δ::kanMX4/rec8-14D-GFP::TRP1::rec8Δ::kanMX4, ura3::tetOx224::URA3/ura3, leu2::*P_{URA3}*-tetR-tdTomato::LEU2/leu2*
Z15213	*MATa/MATα, ndt80Δ::natMX4/ndt80Δ::natMX4, mnd2Δ::hphMX4/mnd2Δ::hphMX4, trp1::*P_{DMC1}*-CDC5::TRP1/trp1::*P_{DMC1}*-CDC5::TRP1, REC8-GFP::URA3/REC8-GFP::URA3, ura3::tetOx224::URA3/ura3, leu2::*P_{URA3}*-tetR-tdTomato::LEU2/leu2*
Z15215	*MATa/MATα, ndt80::HIS3/ndt80::HIS3, mnd2Δ::kanMX4/mnd2Δ::kanMX4, hrr25-as1::HIS3::hrr25Δ::kanMX4/hrr25-as1::HIS3::hrr25Δ::kanMX4, cdc7-4/cdc7-4, REC8-GFP::TRP1::rec8Δ::kanMX4/REC8-GFP::TRP1::rec8Δ::kanMX4, ura3::tetOx224::URA3/ura3, leu2::*P_{URA3}*-tetR-tdTomato::LEU2/leu2*
Z15433	*MATa/MATα, ndt80::HIS3/ndt80::HIS3, mnd2Δ::hphMX4/mnd2Δ::hphMX4, cdc7-4/cdc7-4, rec8-14D-GFP::TRP1::rec8Δ::kanMX4/rec8-14D-GFP::TRP1::rec8Δ::kanMX4, ura3::tetOx224::URA3/ura3, leu2::*P_{URA3}*-tetR-tdTomato::LEU2/leu2*
Z15434	*MATa/MATα, ndt80::HIS3/ndt80::HIS3, mnd2Δ::hphMX4/mnd2Δ::hphMX4, cdc7-4/cdc7-4, REC8-GFP::TRP1::rec8Δ::kanMX4/REC8-GFP::TRP1::rec8Δ::kanMX4, ura3::tetOx224::URA3/ura3, leu2::*P_{URA3}*-tetR-tdTomato::LEU2/leu2*

Z15435	*MATa/MATα, ndt80::HIS3/ndt80::HIS3, mnd2Δ::hphMX4/mnd2Δ::hphMX4, hrr25-as1::HIS3::hrr25Δ::kanMX4/hrr25-as1::HIS3::hrr25Δ::kanMX4, rec8-14D-GFP::TRP1::rec8Δ::kanMX4/rec8-14D-GFP::TRP1::rec8Δ::kanMX4, ura3::tetOx224::URA3/ura3, leu2::*P_{URA3}*-tetR-tdTomato::LEU2/leu2*
Z15436	*MATa/MATα, ndt80::HIS3/ndt80::HIS3, mnd2Δ::hphMX4/mnd2Δ::hphMX4, hrr25-as1::HIS3::hrr25Δ::kanMX4/hrr25-as1::HIS3::hrr25Δ::kanMX4, REC8-GFP::TRP1::rec8Δ::kanMX4/REC8-GFP::TRP1::rec8Δ::kanMX4, ura3::tetOx224::URA3/ura3, leu2::*P_{URA3}*-tetR-tdTomato::LEU2/leu2*
Z15438	*MATa/MATα, rec8-14D-GFP::LEU2::rec8Δ::kanMX4/rec8-14D-GFP::LEU2::rec8Δ::kanMX4, PDS1-tdTomato::KlTRP1/PDS1-tdTomato::KlTRP1*
Z15439	*MATa/MATα, hrr25-as1::HIS3::hrr25Δ::kanMX4/hrr25-as1::HIS3::hrr25Δ::kanMX4, cdc7-4/cdc7-4, rec8-14D-GFP::LEU2::rec8Δ::kanMX4/rec8-14D-GFP::LEU2::rec8Δ::kanMX4, PDS1-tdTomato::KlTRP1/PDS1-tdTomato::KlTRP1*
Z15443	*MATa/MATα, hrr25-zorro::HIS3::hrr25Δ::kanMX4/hrr25-zorro::HIS3::hrr25Δ::kanMX4, cdc7-4/cdc7-4, REC8-GFP::URA3/REC8-GFP::URA3, PDS1-tdTomato::KlTRP1/PDS1-tdTomato::KlTRP1*
Z15446	*MATa/MATα, hrr25-zorro::HIS3::hrr25Δ::kanMX4/hrr25-zorro::HIS3::hrr25Δ::kanMX4, REC8-GFP::URA3/REC8-GFP::URA3, PDS1-tdTomato::KlTRP1/PDS1-tdTomato::KlTRP1*
Z15523	*MATa/MATα, REC8-ha3::LEU2::rec8Δ::kanMX4/REC8-ha3::LEU2::rec8Δ::kanMX4, RTS1-GFP::kanMX4/RTS1-GFP::kanMX4, MTW1-mCherry::hphMX4/MTW1-mCherry::hphMX4*
Z15526	*MATa/MATα, rec8-24A-ha3::LEU2::rec8Δ::kanMX4/rec8-24A-ha3::LEU2::rec8Δ::kanMX4, RTS1-GFP::kanMX4/RTS1-GFP::kanMX4, MTW1-mCherry::hphMX4/MTW1-mCherry::hphMX4*
Z15538	*MATa/MATα, ama1Δ::natMX4/ama1Δ::natMX4, cdc5::*P_{SCC1}*-CDC5::kanMX4/cdc5::*P_{SCC1}*-CDC5::kanMX4, REC8-GFP::URA3/REC8-GFP::URA3, PDS1-tdTomato::KlTRP1/PDS1-tdTomato::KlTRP1*
Z15612	*MATa/MATα, ama1Δ::natMX4/ama1Δ::natMX4, cdc5::*P_{SCC1}*-CDC5::kanMX4/cdc5::*P_{SCC1}*-CDC5::kanMX4, HRR25::HIS3::hrr25Δ::kanMX4/HRR25::HIS3::hrr25Δ::kanMX4, REC8-GFP::URA3/REC8-GFP::URA3, PDS1-tdTomato::KlTRP1/PDS1-tdTomato::KlTRP1*
Z15614	*MATa/MATα, ama1Δ::natMX4/ama1Δ::natMX4, cdc5::*P_{SCC1}*-CDC5::kanMX4/cdc5::*P_{SCC1}*-CDC5::kanMX4, hrr25-as1::HIS3::hrr25Δ::kanMX4/hrr25-as1::HIS3::hrr25Δ::kanMX4, REC8-GFP::URA3/REC8-GFP::URA3, PDS1-tdTomato::KlTRP1/PDS1-tdTomato::KlTRP1*
Z15615	*MATa/MATα, ama1Δ::natMX4/ama1Δ::natMX4, hrr25-as1::HIS3::hrr25Δ::kanMX4/hrr25-as1::HIS3::hrr25Δ::kanMX4, REC8-GFP::URA3/REC8-GFP::URA3, PDS1-tdTomato::KlTRP1/PDS1-tdTomato::KlTRP1*
Z15617	*MATa/MATα, REC8-ha3::LEU2::rec8Δ::kanMX4/REC8-ha3::LEU2::rec8Δ::kanMX4, PDS1-tdTomato::KlTRP1/PDS1-tdTomato::KlTRP1, lys2::tetOx240::URA3/lys2::tetOx240::URA3, leu2::*P_{URA3}*-tetR-GFP::LEU2/leu2::*P_{URA3}*-tetR-GFP::LEU2*
Z15642	*MATa/MATα, esp1-2/esp1-2, rec8-14D-GFP::LEU2::rec8Δ::kanMX4/rec8-14D-GFP::LEU2::rec8Δ::kanMX4, PDS1-tdTomato::KlTRP1/PDS1-tdTomato::KlTRP1, CNM67-tdTomato::natMX4/CNM67-tdTomato::natMX4*
Z15703	*MATa/MATα, ama1Δ::natMX4/ama1Δ::natMX4, cdc7-4/cdc7-4, HRR25::HIS3::hrr25Δ::kanMX4/HRR25::HIS3::hrr25Δ::kanMX4, REC8-GFP::URA3/REC8-GFP::URA3, PDS1-tdTomato::KlTRP1/PDS1-tdTomato::KlTRP1*
Z15704	*MATa/MATα, ama1Δ::natMX4/ama1Δ::natMX4, cdc7-4/cdc7-4, hrr25-as1::HIS3::hrr25Δ::kanMX4/hrr25-as1::HIS3::hrr25Δ::kanMX4, REC8-GFP::URA3/REC8-GFP::URA3, PDS1-tdTomato::KlTRP1/PDS1-tdTomato::KlTRP1*
Z15787	*MATa/MATα, spo11Δ::HIS3MX6/spo11Δ::HIS3MX6, REC8-ha3::LEU2::rec8Δ::kanMX4/REC8, PDS1-tdTomato::KlTRP1/PDS1-tdTomato::KlTRP1, lys2::tetOx240::URA3/lys2, leu2::*P_{URA3}*-tetR-GFP::LEU2/leu2*

Z15788	*MATa/MATα, spo11Δ::HIS3MX6/spo11Δ::HIS3MX6, rec8-24A-ha3::LEU2::rec8Δ::kanMX4/REC8, PDS1-tdTomato::KlTRP1/PDS1-tdTomato::KlTRP1, lys2::tetOx240::URA3/lys2, leu2::*P_{URA3}*-tetR-GFP::LEU2/leu2*
Z15789	*MATa/MATα, spo11Δ::HIS3MX6/spo11Δ::HIS3MX6, REC8-ha3::LEU2::rec8Δ::kanMX4/REC8-ha3::LEU2::rec8Δ::kanMX4, PDS1-tdTomato::KlTRP1/PDS1-tdTomato::KlTRP1, lys2::tetOx240::URA3/lys2, leu2::*P_{URA3}*-tetR-GFP::LEU2/leu2*
Z15790	*MATa/MATα, spo11Δ::HIS3MX6/spo11Δ::HIS3MX6, rec8-24A-ha3::LEU2::rec8Δ::kanMX4/rec8-24A-ha3::LEU2::rec8Δ::kanMX4, PDS1-tdTomato::KlTRP1/PDS1-tdTomato::KlTRP1, lys2::tetOx240::URA3/lys2, leu2::*P_{URA3}*-tetR-GFP::LEU2/leu2*
Z15849	*MATa/MATα, ama1Δ::kanMX4/ama1Δ::kanMX4*
Z15850	*MATa/MATα, ama1Δ::natMX4/ama1Δ::natMX4, cdc5::*P_{SCC1}*-CDC5::kanMX4/cdc5::*P_{SCC1}*-CDC5::kanMX4*
Z15887	*MATa/MATα, spo11Δ::HIS3MX6/spo11Δ::HIS3MX6, cdc7-4/cdc7-4, hrr25-as1::HIS3::hrr25Δ::kanMX4/hrr25-as1::HIS3::hrr25Δ::kanMX4, PDS1-myc18::KlTRP1/PDS1-myc18::KlTRP1, *P_{REC8}*-SCC1-ha3::LEU2::rec8Δ::kanMX4/*P_{REC8}*-SCC1-ha3::LEU2::rec8Δ::kanMX4*
Z15888	*MATa/MATα, spo11Δ::HIS3MX6/spo11Δ::HIS3MX6, cdc7-4/cdc7-4, hrr25-as1::HIS3::hrr25Δ::kanMX4/hrr25-as1::HIS3::hrr25Δ::kanMX4, PDS1-myc18::KlTRP1/PDS1-myc18::KlTRP1*
Z15948	*MATa/MATα, ndt80::HIS3/ndt80::HIS3, mnd2Δ::hphMX4/mnd2Δ::hphMX4, bob1::natMX4/bob1::natMX4, REC8-GFP::URA3/REC8-GFP::URA3, ura3::tetOx224::URA3/ura3, leu2::*P_{URA3}*-tetR-tdTomato::LEU2/leu2*
Z15949	*MATa/MATα, ndt80::HIS3/ndt80::HIS3, mnd2Δ::hphMX4/mnd2Δ::hphMX4, bob1::natMX4/bob1::natMX4, cdc7Δ::kanMX4/cdc7Δ::kanMX4, REC8-GFP::URA3/REC8-GFP::URA3, ura3::tetOx224::URA3/ura3, leu2::*P_{URA3}*-tetR-tdTomato::LEU2/leu2*
Z16025	*MATa/MATα, REC8-ha3::LEU2::rec8Δ::kanMX4/REC8, ubr1Δ::KlTRP1/ubr1Δ::KlTRP1, ndt80Δ::natMX4/ndt80Δ::natMX4*
Z16026	*MATa/MATα, REC8-ha3::LEU2::rec8Δ::kanMX4/REC8, ubr1Δ::KlTRP1/ubr1Δ::KlTRP1, ndt80Δ::natMX4/ndt80Δ::natMX4, mnd2Δ::hphMX4/mnd2Δ::hphMX4, hrr25-as1::HIS3::hrr25Δ::kanMX4/hrr25-as1::HIS3::hrr25Δ::kanMX4*
Z16027	*MATa/MATα, REC8-ha3::LEU2::rec8Δ::kanMX4/REC8, ubr1Δ::KlTRP1/ubr1Δ::KlTRP1, ndt80Δ::natMX4/ndt80Δ::natMX4, mnd2Δ::hphMX4/mnd2Δ::hphMX4, cdc7-4/cdc7-4*
Z16135	*MATa/MATα, REC8-ha3::LEU2::rec8Δ::kanMX4/REC8, ubr1Δ::KlTRP1/ubr1Δ::KlTRP1, ndt80Δ::natMX4/ndt80Δ::natMX4, mnd2Δ::hphMX4/mnd2Δ::hphMX4, cdc7-4/cdc7-4, hrr25-as1::HIS3::hrr25Δ::kanMX4/hrr25-as1::HIS3::hrr25Δ::kanMX4*
Z16150	*MATa/MATα, REC8-GFP::LEU2::rec8Δ::kanMX4/REC8-GFP::LEU2::rec8Δ::kanMX4, PDS1-tdTomato::KlTRP1/PDS1-tdTomato::KlTRP1, CNM67-tdTomato::natMX4/CNM67-tdTomato::natMX4, sgo1::kanMX6::*P_{CLB2}*-ha3-SGO1/sgo1::kanMX6::*P_{CLB2}*-ha3-SGO1*
Z16151	*MATa/MATα, rec8-24A-GFP::LEU2::rec8Δ::kanMX4/rec8-24A-GFP::LEU2::rec8Δ::kanMX4, PDS1-tdTomato::KlTRP1/PDS1-tdTomato::KlTRP1, CNM67-tdTomato::natMX4/CNM67-tdTomato::natMX4, sgo1::kanMX6::*P_{CLB2}*-ha3-SGO1/sgo1::kanMX6::*P_{CLB2}*-ha3-SGO1*
Z16157	*MATa/MATα, REC8-GFP::LEU2::rec8Δ::kanMX4/REC8-GFP::LEU2::rec8Δ::kanMX4, PDS1-tdTomato::KlTRP1/PDS1-tdTomato::KlTRP1, sgo1::kanMX6::*P_{CLB2}*-ha3-SGO1/sgo1::kanMX6::*P_{CLB2}*-ha3-SGO1, cdc7-4/cdc7-4, hrr25-as1::HIS3::hrr25Δ::kanMX4/hrr25-as1::HIS3::hrr25Δ::kanMX4*
Z16178	*MATa/MATα, REC8-GFP::LEU2::rec8Δ::kanMX4/REC8-GFP::LEU2::rec8Δ::kanMX4, PDS1-tdTomato::KlTRP1/PDS1-tdTomato::KlTRP1, sgo1::kanMX6::*P_{CLB2}*-ha3-SGO1/sgo1::kanMX6::*P_{CLB2}*-ha3-SGO1*
Z16220	*MATa/MATα, rec8-24A-ha3::LEU2::rec8Δ::kanMX4/REC8, ubr1Δ::KlTRP1/ubr1Δ::KlTRP1, ndt80::HIS3/ndt80::HIS3, mnd2Δ::CaURA3/mnd2Δ::CaURA3*

Z16221	*MATa/MATα, rec8-24A-ha3::LEU2::rec8Δ::kanMX4/REC8, ubr1Δ::KlTRP1/ubr1Δ::KlTRP1, ndt80::HIS3/ndt80::HIS3*
Z16253	*MATa/MATα, cdc5::P_{SCC1}-CDC5::kanMX4/cdc5::P_{SCC1}-CDC5::kanMX4, REC8-GFP::URA3/REC8-GFP::URA3, PDS1-tdTomato::KlTRP1/PDS1-tdTomato::KlTRP1*
Z16346	*MATa/MATα, ama1Δ::natMX4/ama1Δ::natMX4, REC8-GFP::URA3/REC8-GFP::URA3, PDS1-tdTomato::KlTRP1/PDS1-tdTomato::KlTRP1*
Z16490	*MATa/MATα, ndt80Δ::natMX4/ndt80Δ::natMX4*
Z16491	*MATa/MATα, ndt80Δ::natMX4/ndt80Δ::natMX4, cdc7-4/cdc7-4*
Z16492	*MATa/MATα, ndt80Δ::natMX4/ndt80Δ::natMX4, hrr25-as1::HIS3::hrr25Δ::kanMX4/hrr25-as1::HIS3::hrr25Δ::kanMX4*
Z16493	*MATa/MATα, ndt80Δ::natMX4/ndt80Δ::natMX4, cdc7-4/cdc7-4, hrr25-as1::HIS3::hrr25Δ::kanMX4/hrr25-as1::HIS3::hrr25Δ::kanMX4*

*K8965 and K8967 are haploid W303 strains with the background: *ade2-1 trp1-1, can1-100 leu2-3,112 his3-11,15 ura3*. All other strains are diploid SK1 strains with the background: *ho::LYS2/ho::LYS2, lys2/lys2, ade2Δ::hisG/ade2Δ::hisG, trp1Δ::hisG/trp1Δ::hisG, leu2Δ::hisG/leu2Δ::hisG*.

SUPPLEMENTAL REFERENCES

Altschul, S.F., Madden, T.L., Schäffer, A.A., Zhang, J., Zhang, Z., Miller, W., and Lipman, D.J. (1997). Gapped BLAST and PSI-BLAST: a new generation of protein database search programs. Nucleic Acids Res. *25*, 3389-3402.

Baker, N.A., Sept, D., Joseph, S., Holst, M.J., and McCammon, J.A. (2001). Electrostatics of nanosystems: application to microtubules and the ribosome. Proc. Natl. Acad. Sci. USA *98*, 10037-10041.

Hardy, C. F., Dryga, O., Seematter, S., Pahl, P. M., and Sclafani, R. A. (1997). mcm5/cdc46-bob1 bypasses the requirement for the S phase activator Cdc7p. Proc Natl Acad Sci U S A *94*, 3151-3155.

Jinek, M., Rehwinkel, J., Lazarus, B.D., Izaurralde, E., Hanover, J.A., and Conti, E. (2004). The superhelical TPR-repeat domain of O-linked GlcNAc transferase exhibits structural similarities to importin alpha. Nat. Struct. Mol. Biol. *11*, 1001-1007.

Karpenahalli, M.R., Lupas, A.N., and Söding, J. (2007). TPRpred: a tool for prediction of TPR-, PPR- and SEL1-like repeats from protein sequences. BMC Bioinformatics *8*:2.

Matos, J., Lipp, J.J., Bogdanova, A., Guillot, S., Okaz, E., Junqueira, M., Shevchenko, A., and Zachariae, W. (2008). Dbf4-dependent Cdc7 kinase links DNA replication to the segregation of homologous chromosomes in meiosis I. Cell *135*, 662-678.

Petronczki, M., Matos, J., Mori, S., Gregan, J., Bogdanova, A., Schwickart, M., Mechtler, K., Shirahige, K., Zachariae, W., and Nasmyth, K. (2006). Monopolar attachment of sister kinetochores at meiosis I requires casein kinase 1. Cell *126*, 1049-1064.

Söding, J. (2005). Protein homology detection by HMM-HMM comparison. Bioinformatics *21*, 951-960.
